# Restrictive Eating Patterns and Eating Disorder Risk in Female Ballet Dancers: Exploratory Associations with Self-Reported Workplace Bullying and Possible Mobbing

**DOI:** 10.3390/nu18193191

**Published:** 2026-09-27

**Authors:** Marius Baranauskas, Ingrida Kupčiūnaitė, Jurgita Lieponienė, Rimantas Stukas

**Affiliations:** 1 Faculty of Biomedical Sciences, State Higher Education Institution Panevėžys College, 35200 Panevėžys, Lithuania; ingrida.kupciunaite@panko.lt (I.K.); jurgita.lieponiene@panko.lt (J.L.); 2 Department of Public Health, Institute of Health Sciences, Faculty of Medicine, Vilnius University, 01513 Vilnius, Lithuania; rimantas.stukas@mf.vu.lt

**Keywords:** ballet dancers, EAT-26, eating disorder risk, HSE indicator tool, mental health, nutritional health, occupational stress, oral control, public health

## Abstract

Background/Objectives: While elevated risks of disordered eating attitudes (DEAs) among ballet dancers are well-documented, the underlying organizational and psychosocial factors in their work environment have received less attention. The aim of the present study was to assess the proportion and distinct behavioral patterns of eating disorder (ED) risk among female ballet dancers compared with university students from high-risk disciplines, and to identify workplace stressors associated with clinically relevant ED symptoms. Methods: A cross-sectional study was conducted among professional ballet dancers (*n* = 117). For comparison, a cohort of higher education students from high-risk disciplines, specifically Medical and Nutritional Sciences as well as Arts (*n* = 260), was recruited. DEAs were assessed using the Eating Attitudes Test-26 (EAT-26). Perceived occupational stress among ballet dancers was quantified using the Health and Safety Executive (HSE) Indicator Tool. Results: When applying the screening threshold (EAT-26 ≥ 20), 35.9% of ballet dancers had clinically relevant ED symptoms, notably surpassing student groups (approximately 25%) (*p* = 0.003). The discriminant function analysis identified distinct ED behavioral profiles. Among students, ED symptoms were predominantly associated with Factor B (Bulimia and Food Preoccupation). In contrast, 49.1% of ballet dancers were classified within the highest risk category for Factor O (Oral Control), indicating rigid dietary restriction. The observed association between perceived occupational stress, specifically related to self-reported workplace bullying (β 3.5, 95% confidence interval (CI): 0.1; 7.1, *p* = 0.049) and possible mobbing (β 4.4, 95% CI: 0.1; 8.7, *p* = 0.045), and the expression of clinically relevant ED symptomatology among ballet dancers can be conceptualized within a hypothetical socio-neuro-behavioral framework. Conclusions: The ballet-specific restrictive eating pattern (Factor O) tracks as a potential behavioral coping response to navigate rigid theatrical work environments. Multi-level interventions that address workplace mental health are needed to support healthy eating patterns and metabolic balance among ballet dancers.

## 1. Introduction

Eating disorders (EDs), including anorexia nervosa, bulimia nervosa, and binge-eating disorder, are a critical global public health concern characterized by severe physiological impairment and profound psychiatric comorbidity [1]. According to the Diagnostic and Statistical Manual of Mental Disorders, Fifth Edition, Text Revision (DSM-5-TR), EDs are conceptualized as persistent disturbances in eating behavior that fundamentally alter eating habits and seriously impair both physical health and psychosocial functioning [2].

Recent epidemiological data indicate that the lifetime prevalence of EDs in the general population ranges from 2.5% to more than 8%, with a marked increase among young adults over the past decade [3,4]. These high and persistent prevalence rates across vulnerable demographic groups internationally make EDs a major public health challenge [1]. Clinically relevant ED symptoms can co-occur with severe metabolic disorders, energy deficiency, and somatic complications, such as cardiac arrhythmia, electrolyte imbalance, and multiple organ failure [3,4]. Given these multi-system consequences associated with the expression of ED symptoms, effective prevention and management of ED symptomology require a clear understanding of how environmental stressors intersect with individual ED risk profiles expressed in behavioral patterns [5,6,7].

Although subclinical disordered eating attitudes (DEAs) are increasingly widespread across the general population [8], they are particularly prevalent in specific target populations exposed to extreme environmental pressures [9]. For example, studies of university students, particularly those involved in highly competitive and stressful disciplines, namely Arts, Medicine, Health Sciences, and Nutritional Sciences, have reported alarming DEA proportions that frequently exceed 20–25% [10,11,12].

On the other hand, dancers appear to be considerably vulnerable to EDs. Compared with the general population, ballet dancers have been reported to occupy a highly vulnerable occupational position facing up to a threefold higher risk of exhibiting clinically relevant ED symptomatology [13,14,15,16,17,18,19]. Across several decades, this increased vulnerability of ballet dancers has been linked to the occurrence of ED symptoms and traditionally attributed to individual psychopathological personality traits such as perfectionism [15,18,20], body image distortion [17,21], or the broader aesthetic standards of the art form [19,22,23,24].

In ballet environments, this vulnerability frequently co-occurs with the intensive appearance evaluation, body surveillance, and continuous exposure to idealized physical standards. The interplay between appearance-related social evaluation and heightened self-awareness is often closely associated with body dissatisfaction and social physique anxiety among performing artists. This conceptual link aligns with broader psychological frameworks suggesting that heightened appearance-based social evaluation and external validation pressures are significantly correlated with negative body-related psychological outcomes, including increased body dissatisfaction and distress [25]. In the context of professional ballet, these heightened aesthetic and social evaluation pressures may create a psychological climate where individual vulnerabilities are reinforced, further intersecting with the adoption of restrictive eating patterns.

However, interventions that have mainly focused on the individual have failed to significantly reduce the proportion of EDs in ballet populations [13,18,20,26]. Professional ballet is not only an artistic pursuit but also a highly rigid, socio-economically precarious, and intensely hierarchical occupational ecosystem [15,18,22,27,28,29,30]. Therefore, modern research methods should also consider multi-level potential risk factors that may be associated with the nutritional health of ballet dancers.

Structural and organizational occupational stressors [15,31], generally classified as psychosocial strain, may contribute to behavioral and somatic pathologies but remain under-researched in ballet populations [32]. Understanding complex interactions among organizational systems, perceived psychological stress, and dietary disturbances among ballet dancers is therefore particularly relevant in the present case [26]. The institutional psychosocial strain inherent in the daily routines of dancers may manifest as interpersonal friction, including self-reported workplace bullying and potential mobbing, with potential implications for physiological functions and nutritional status.

From a neurobiological perspective and based on previous theoretical models, chronic psychological harassment may be conceptualized as an unpredictable, low-control survival threat [33]. Perceived chronic psychological stress is theoretically hypothesized to involve sustained activation of subcortical emotional networks, particularly the amygdala, and has been linked in the literature with a prolonged secretion of cortisol and corticotropin-releasing hormone or hypothalamic–pituitary–adrenal axis dysregulation [34,35]. When personal safety, institutional status, and financial security are systematically threatened, especially among lower-ranking professionals (e.g., *corps de ballet* dancers and *coryphées*) and those with low incomes, the brain’s prefrontal cortex is hypothesized to engage in compensatory top-down cognitive control to restore a sense of control or personal autonomy [33,36]. In this framework, when their professional environment is perceived as difficult to control, ballet dancers may attempt to enforce absolute, rigid, and unyielding control over their dietary intake and physical body [37].

University students under heavy academic strain and intense psychosocial demands typically encounter examination-related and cyclical psychological stress. This academic stress framework, structurally and neurobiologically, differs from hostile hierarchical pressures found in professional theater environments and may therefore be associated with distinct pathways of ED symptom expression [10]. Previous scientific evidence suggests that students at risk for EDs may frequently exhibit bulimic tendencies or emotional food preoccupation [11] due to neuroendocrine shifts [35,38], whereas vulnerable ballet dancers may exhibit a highly restrictive eating pattern defined by extreme behavioral inhibition and rigid food restriction reflected in oral control [13,14,16,23,37]. This distinct eating profile characterized by oral control can also pose severe nutritional risks resulting in low energy availability, relative energy deficiency in sport, and reduced bone mineral density [39]. As ballet-related psychological stress has often been treated as a general consequence of physical workload and choreographic demands [27], rather than examined in relation to localized institutional strain, the present study addressed this gap by using a two-stage cross-sectional design.

The primary aim of this study was to evaluate the proportion and distinct behavioral profiles of ED risk among female ballet dancers and university students from high-risk disciplines, and to identify the specific item-level organizational stressors associated with these behavioral characteristics. To guide our statistical analyses, the following directional hypotheses were tested:

**Primary Hypothesis** **(H1).** *Female ballet dancers exhibit a significantly higher proportion of clinically relevant ED symptoms and a distinct pattern of restrictive behavior characterized by dominant oral control (Factor O), whereas student cohorts exhibit predominantly bulimic and food-preoccupation tendencies (Factor B).*

**Secondary Hypothesis** **(H2).** *Self-reported exposure to workplace bullying and possible mobbing within the institutional hierarchy is significantly associated with higher overall ED symptomatology and greater oral control (Factor O) among female ballet dancers.*

## 2. Materials and Methods

### 2.1. Study Design, Participants and Data Collection

This study employed a two-phase analytical approach. First, a comparative cross-sectional study design was conducted in the Republic of Lithuania between September 2024 and April 2025 to evaluate the characteristics of potential ED symptomatology across the target cohorts. Female ballet dancers were recruited from the Lithuanian National Opera and Ballet Theatre, Kaunas State Musical Theatre, and Klaipėda State Musical Theatre. The student reference group was intentionally selected from Arts, Nutritional Sciences, and Medical Science programs because these cohorts represent a known high-risk population for ED symptomatology. This comparison group was integrated in order to evaluate whether professional ballet dancers exhibit an elevated ED risk profile that statistically exceeds that of another already vulnerable cohort. More specifically, a reference group of female higher education students was established in the major Lithuanian cities of Vilnius, Kaunas, and Klaipėda.

To ensure sufficient statistical power, based on the eligible target populations of professional ballet dancers (*N* = 139) and higher education students (*N* = 22,234), minimum required sample sizes were calculated using the web-based freeware OpenEpi version 3.01 [40]. At a 95% confidence level and a 7% margin of error, and assuming a hypothesized outcome frequency of 50% to achieve maximum sample variability, the required baseline threshold sample sizes were *n* = 82 for the ballet cohort and *n* = 195 for the student cohort. Using non-probability purposive sampling based on these predefined criteria, the ballet cohort yielded 117 analyzed cases from 139 eligible professionals, corresponding to an active response rate of 84.2%. For the student cohort, the final sample comprised 260 participants who fully met the predefined academic-discipline criteria following the broader screening process. The student cohort yielded an initial response rate of 1.17%.

The explicit inclusion criteria for the professional ballet dancer cohort were as follows: (1) an active, paid, fixed-term employment contract with a recognized national dance company, such as one of the Lithuanian national theaters; (2) completion of multi-year professional vocational or higher education training in dance; (3) active employment as a professional ballet dancer; (4) age > 18 years; and (5) no self-reported history of clinical treatment for EDs within the previous 12 months. Correspondingly, the student comparison group was required to meet the following eligibility criteria: (1) current enrollment in a Lithuanian higher education institution; (2) study in Medical and Health Sciences, specifically including Medical and Nutritional sub-disciplines, or Arts and Humanities; (3) age > 18 years; and (4) no clinical treatment for EDs during the previous 12 months.

As shown in the study flowchart (Figure 1), exclusion criteria were strictly applied during recruitment for both cohorts. The exclusion criteria for the professional ballet dancer sample were as follows: (1) male sex (*n* = 12); (2) non-Lithuanian nationality (*n* = 1); (3) hospitalization for an ED within the previous 12 months (*n* = 1); or (4) formal decline to participate in the study (*n* = 8). Student candidates for the reference student group were excluded if they met any of the following criteria: (1) male sex (*n* = 20); (2) enrollment in academic programs outside the predefined Medical, Nutritional, and Arts Sciences domains (*n* = 189); (3) non-Lithuanian nationality (*n* = 15); (4) history of hospitalization for an ED during the previous year (*n* = 3); and (5) non-response to the electronic survey (*n* = 21,747).

Regarding missing data handling, a complete-case analysis (listwise deletion) was strictly implemented. Since the small sample size of recruited males would severely compromise the validity and statistical power of subgroup analyses, thereby precluding reliable multi-group comparisons, male participants (total *n* = 32; *n* = 12 ballet dancers and *n* = 20 students) from both studied cohorts were excluded from the final analysis to ensure sample homogeneity and preserve statistical power.

Finally, most ballet dancers were employed at the Lithuanian National Opera and Ballet Theatre (*n* = 76, 65.0%), followed by Kaunas State Musical Theatre (*n* = 21, 17.9%) and Klaipėda State Musical Theatre (*n* = 20, 17.1%). In the comparison group, 61.2% of the students lived in the capital city of Vilnius (*n* = 159), while the remaining participants lived mostly in Kaunas (*n* = 42, 16.2%) and Klaipėda (*n* = 59, 22.7%), and in other major urban centers of Lithuania.

Data were collected using a structured, anonymous online questionnaire hosted on the ‘Apklausa’ electronic survey platform (Apklausa.lt, Vilnius, Lithuania). The survey link was distributed to the professional ballet dancers through the institutional communication channels of the participating theaters. Students comprising a comparison group were recruited through university virtual learning environments and institutional networks.

In the first phase of the present study, significantly higher Eating Attitudes Test-26 (EAT-26) [41,42] scores were confirmed among ballet dancers. The second phase focused exclusively on the ballet cohort to examine occupational stress using the HSE Management Standards Indicator Tool [43]. As shown in Figure 2, the student comparison cohort was excluded from this stage because the participants were full-time students and were not engaged in formal professional employment.

The HSE Indicator Tool was specifically validated to measure organizational and workplace stressors, including ‘Managerial support’, ‘Peer support’, and ‘Relationships’. Applying this instrument to a non-working student population would lack contextual validity. In contrast, since professional ballet dancers represented a unique, highly rigid occupational sample characterized by intense physical demands, strict institutional hierarchies, and contract insecurity, the second phase of the study focused on identifying specific occupational stressors associated with abnormal eating behaviors within the ballet cohort of our study.

### 2.2. Measures and Instruments

#### 2.2.1. Sociodemographic Characteristics and Eating Attitudes Test (EAT-26)

A structured background questionnaire was used to collect relevant sociodemographic and institutional characteristics. For the full study sample, primary variables included age, monthly disposable income, and marital status. For the professional ballet cohort, advanced occupational variables were extensively mapped, encompassing the specific institutional hierarchy rank (*corps de ballet*, soloists, *coryphée*, or principal dancer), professional seniority, daily practice duration, and annual number of public performances during the season from September to June. For the student comparison group, academic variables included city of residence, type of educational institution, degree level (undergraduate versus graduate/doctoral), and year of study.

Potential ED risk and eating behavioral patterns were assessed using the standardized EAT-26 [41,42,44]. Participants rated each item on a 6-point Likert scale. A cumulative EAT-26 score threshold ≥ 20 was established to define clinically relevant potential ED risk [45,46]. In addition to the total cumulative scale mapping, the instrument was stratified into three validated subscales: Dieting (Factor D), Bulimia and Food Preoccupation (Factor B), and Oral Control (Factor O) [47]. The ED psychometric Factor D was closely linked to a distorted body image; Factor B was associated with bulimic behaviors, loss of control around eating, and thoughts of bingeing; and Factor O was related to restrictive eating patterns without bulimia. In the present study, the internal consistency of the EAT-26 instrument was calculated across the entire pooled sample (*n* = 377) and showed excellent reliability, yielding a total Cronbach’s alpha (α) coefficient of 0.918. Item-level internal consistency matrices and subscale-specific reliability estimates are presented in Table S1.

#### 2.2.2. Psychosocial Working Conditions: The HSE Indicator Tool

Occupational stress and psychosocial working conditions within the theater hierarchy were measured using the 35-item Health and Safety Executive (HSE) Indicator Tool (Lithuanian version) [43,48,49,50]. The HSE scale systematically maps seven core dimensions of workplace strain: Demands (workload, work structure and work environment), Control (employee impact on work performance), Managerial support and Peer support (encouragement, help and supply provided by the institution, line managers and colleagues), Relationships (promotion of positive relationships and the prevention of conflict and unacceptable behavior), Role (clarity of employee’s role within the organization and absence of role conflict), and Change (management and communication of organizational change). The ‘Relationships’ subscale also serves as an exploratory and indirect proxy for workplace bullying and possible mobbing, defined here as exposure to systematic personal harassment and negative interpersonal behaviors within the organizational hierarchy. This subscale provides a valid indicator for exploratory screening because its standardized items explicitly evaluate severe relational abuse, specifically via item Q5 (‘I am subject to personal harassment in the form of unkind words or behavior’, screening possible mobbing) and item Q21 (‘I am subject to bullying at work’, screening bullying) (Table S2). All HSE items were scored using standard 5-point Likert scales, with higher scores reflecting more favorable psychosocial conditions. Because this occupational health questionnaire evaluates specific professional environments [51], it was administered exclusively to the professional ballet dancer cohort, as the reference student subgroups held no occupational status. The internal consistency of the total HSE Indicator Tool within the ballet dancer sample (*n* = 117) was exceptionally high, yielding a total Cronbach’s α = 0.896. The subscale-specific coefficients remained highly satisfactory, ranging from 0.877 for the ‘Relationships’ domain to 0.908 for the ‘Managerial support’ domain, too. The item-level reliability values are presented in Table S2.

#### 2.2.3. Conceptual Neurobiological Framework and Brain Mapping

To contextualize the study findings within a theoretical neurobiological framework, a human brain color map was generated using the Science and Research online plotting platform (SRplot, version 2023; Science and Research, Hangzhou, China) [52]. The visualization used the cerebroViz R package (version 1.0.0; The University of Iowa, Iowa City, IA, USA) within R version 4.3.2 (R Foundation for Statistical Computing, Vienna, Austria) to project standardized regional values onto vector graphic templates of the human brain [53]. Specific structural localization and region definitions were based on BrainSpan anatomical atlas configurations (version 1.0; Allen Institute for Brain Science, Seattle, WA, USA), providing standardized mapping across discrete cortical and subcortical regions [54].

Based on current functional neuroimaging (fMRI) theoretical frameworks regarding restrictive eating patterns, theoretical and speculative weights were systematically assigned to specific regions of interest to visually map literature-guided hypotheses. Executive control networks, including the dorsolateral prefrontal cortex and medial prefrontal cortex, were modeled with higher theoretical values (5.2–5.4) to conceptualize the rigid top-down cognitive control potentially associated with Factor O (oral control) traits. Subcortical threat-processing regions, including the amygdala, were assigned elevated theoretical values (1.2) to represent hypothesized activation pathways related to self-reported workplace bullying and possible mobbing. For anatomical and topographic contrast, baseline values (0.1) were assigned to sensorimotor regions, such as the cerebellum. This modeling serves exclusively as a conceptual visualization of pathways derived from theoretical frameworks and does not represent empirical neuroimaging or physiological data measured in the present sample. This visualization and its underlying framework are integrated and utilized exclusively within the Discussion section (Section 4.3) to guide exploratory hypothesis generation and contextualize the identified statistical associations.

### 2.3. Statistical Data Analysis

Statistical data analyses were conducted using the Statistical Package for the Social Sciences (IBM SPSS Statistics), version 25.0 for Windows (IBM Corp., Armonk, NY, USA). Data visualizations were created using IBM SPSS, LibreOffice version 7.6.4 (The Document Foundation, Berlin, Germany), and the open-access web tool SRplot [52].

Prior to the main statistical analyses, the Shapiro–Wilk test was performed to evaluate the normality of all continuous variables. Categorical data were summarized using frequency distribution tables and expressed as frequencies and percentages. Means and corresponding 95% confidence intervals (CIs) were used to summarize numerical outcomes, measures of central tendency and dispersion for each analyzed variable.

For bivariate analyses, differences in categorical variables (age group, workplace, dancer rank, education and income levels, marital status and seniority) between the subgroups of ballet dancers with different EAT-26 risk levels were assessed using the chi-square (χ^2^) test, coupled with odds ratios (ORs). To compare measures of central tendency between the groups under analysis, the independent-samples *t*-test was applied, and Cohen’s d was calculated as an effect-size measure. Following Cohen [55], the effect sizes were interpreted as small (0.2 ≤ d < 0.5), moderate (0.5 ≤ d < 0.8), or large (d ≥ 0.8).

A stepwise linear discriminant analysis (LDA) [56] was used to evaluate the classification and statistical grouping of study participants according to the predominant expression of the three EAT-26 subscales: Dieting (Factor D), Bulimia and Food Preoccupation (Factor B), and Oral Control (Factor O). Prior to conducting the analysis, the core assumptions of LDA were evaluated: the normality of the independent variables was assessed via Shapiro–Wilk tests and visual inspection of Q-Q plots, and the homogeneity of the variance-covariance matrices was assessed using Box’s M test. The LDA model was established sequentially by entering specific independent variables with significant discriminatory power between the studied cohorts. The statistical significance of the derived canonical discriminant functions was evaluated using Wilks’ lambda (Λ) and chi-square (χ^2^) tests. Overall classification accuracy was assessed by calculating the percentage of correctly grouped cases across the models. To evaluate the generalizability of the classification performance and account for potential overfitting, leave-one-out cross-validation was performed. A classification matrix was then used to compute the precise proportion of correct assignments, group-specific sensitivity, and specificity, as well as to evaluate potential cross-cohort statistical overlaps.

For multivariate modeling, multiple linear regression models were constructed to evaluate the association between the HSE core occupational stress dimensions (independent variables) and total EAT-26 scores or subscale domains (dependent variables) within the professional ballet dancer cohort. All linear regression models were adjusted for institutional rank, monthly income, and marital status, which served as covariates. These specific covariates were empirically selected based on preliminary bivariate analysis of all collected sociodemographic and institutional variables. Only variables demonstrating statistically significant baseline associations with either primary ED risk profiles or perceived psychosocial workplace stressors (HSE subscales) were retained in the multivariable regression models to strictly control for intra-group confounding.

Additionally, the coefficient of determination (R^2^) and the F-statistic were calculated to check the goodness of fit of each regression model. Prior to modeling, all foundational linear regression assumptions were systematically assessed: linearity and homoscedasticity were evaluated through residual distribution checks, multicollinearity was ruled out using a Variance Inflation Factor (VIF) threshold of <2.5, and influential observations were evaluated using Cook’s distance, with no cases exceeding the critical threshold of 1.0. To mitigate the risk of Type I error given the exploratory nature of this study within a specialized cohort (*n* = 117), the multivariable regression analyses were restricted to theoretically and empirically justified blocks, and findings were interpreted with emphasis on the variance explained (R^2^) and the magnitude of associations rather than relying solely on statistical significance based on *p*-values (α = 0.05).

Finally, this cross-sectional study was reported in accordance with the checklist of the Strengthening the Reporting of Observational Studies in Epidemiology (STROBE) guidelines [57].

## 3. Results

### 3.1. Baseline Characteristics and Potential Eating Disorder Risk Distribution

A total of 117 female ballet dancers were enrolled in the study. Their sociodemographic and occupational baseline characteristics are summarized in Table 1. The mean age of the ballet cohort was 23.9 years (95% CI [29.0; 30.9]); 51.3% were aged 20–22 years and 48.7% were aged 23–45 years. Mean professional dance seniority was 7.5 years (95% CI [6.7, 8.4]). In terms of weekly workloads, the cohort reported a mean of 29.9 h (95% CI [29.0, 30.9]) per week of training and dance practice, typically distributed across 5–6 sessions per week ranging from 2.5 to 5 h per day. The annual volume of public performances varied from 20 to 50 per season (September–June). Within the professional hierarchy, 53.0% of the participants were *corps de ballet* dancers, 17.1% were *coryphées*, 21.4% were soloists, and 8.5% were principal dancers. Most participants reported a monthly income of 600–1500 EUR (57.3%), and 70.1% were single or in a relationship, whereas 29.9% were married.

The reference cohort comprised 260 female higher education students from Arts and Nutritional Sciences and Medical Science programs. Their mean age was 21.2 years (95% CI [20.8, 21.6]). In terms of academic distribution, most students were enrolled at universities (85.4%), while 14.6% attended colleges. Monthly disposable income was distributed almost equally between 500–800 EUR (49.2%) and 801–3500 EUR (50.8%). First-year students accounted for 47.3% of the sample, while those in academic years 2–6 made up 52.7%. Finally, the sample demonstrated a symmetrical distribution across academic degrees, with undergraduate (BSc) students representing 50% of the cohort, while graduate (MSc) or doctoral (PhD) students accounted for the remaining 50%.

As shown in Figure 3, a significant difference was observed in the distribution of ED risk proportions across the studied cohorts. Specifically, 35.9% (approximately 1/3) of female ballet dancers met the screening EAT-26 threshold of >20 compared with 25.6% of Art and Nutritional Sciences students and 24.4% of Medical Science students. Thus, clinically relevant ED symptomatology was more frequent among ballet dancers than among either student subgroup.

Ballet dancers also had significantly higher mean total EAT-26 scores than Art and Nutritional Sciences students (d = 0.3, 95% CI: 0.1; 0.6). The differences between the groups were primarily reflected in Factor D (Dieting; d = 0.3, 95% CI: 0.1; 0.4) and Factor O (Oral Control; d = 0.5, 95% CI: 0.3; 0.7), with both subscales demonstrating statistically significant differences.

More detailed crude values for the EAT-26 total and its subscales (Factors D, B, and O) are presented in Table 2.

As the individual EAT-26 subscales contain an unequal number of items, direct comparison of their raw mean scores remains statistically limited and cannot definitively establish which specific ED risk profile dominated within or across the subgroups under analysis. To overcome this limitation of raw scale asymmetry and to isolate the ‘true’ ED profile that distinguishes these cohorts, a canonical discriminant analysis was subsequently performed. This statistical approach standardized the variables simultaneously, allowing for the identification of group-specific eating attitudes configurations based on their combined variance rather than isolated raw mean scores.

### 3.2. Eating Attitudes Profiles and Their Sociodemographic Context

As shown in Figure 4, the initial discriminant model correctly classified 46.1% of the original grouped cases. To evaluate the generalizability of the classification performance, leave-one-out cross-validation yielded an overall cross-validated accuracy rate of 42.8%. Although this statistically exceeded the 33.3% baseline chance level expected under equal-probability classification across three groups, both canonical discriminant functions derived from the stepwise analysis were statistically significant (Function 1: Wilks’ Λ = 0.84, χ^2^(6) = 31.2, *p* < 0.001; Function 2: Wilks’ Λ = 0.95, χ^2^(2) = 6.3, *p* = 0.049).

To assess the classification utility of the model, group-specific sensitivity (correct classification rate) and specificity (correct exclusion rate) were evaluated using the cross-validated classification matrix. For the ballet dancer cohort, the model demonstrated a cross-validated sensitivity of 49.1% and a specificity of 75.2%, indicating that the model correctly identified 49.1% of the ballet-dancer cases and correctly classified 75.2% of non-ballet cases as not belonging to the ballet-dancer cohort.

By contrast, the student cohorts displayed notable boundary overlaps. The Arts and Nutritional Sciences (ANSs) group showed a sensitivity of 49.2% and a specificity of 64.3%. The lowest classification sensitivity was observed in the Medical Science students (MSs) group at 30.0%, although it maintained a specificity of 74.2%, with 41.7% of MSs cases being misclassified as the ANSs group. This directional overlap suggested that the two student cohorts exhibited partially overlapping eating disorder characteristics, predominantly associated with bulimic and food preoccupation traits (Factor B). Taken together, the discriminant analysis suggested that the ballet cohort was characterized by a more prominent expression of oral control (Factor O) traits within a moderate overall statistical separation and with notable boundary overlap among the three cohorts.

Before transitioning this distinct eating attitudes profile of ballet-specific Factor O into a predictive value, it was essential to assess potential intra-group confounding variables within the ballet cohort itself. Secondary bivariate analyses based on the variables shown in Table 1 were therefore conducted to identify individual sociodemographic and occupational characteristics associated with clinically relevant ED symptomatology and to select covariates for subsequent multivariable regression models.

Bivariate analyses comparing the differences between ED risk groups (EAT-26 score ≥ 20 vs. <20) across sociodemographic and occupational characteristics showed a largely homogeneous distribution across most variables, with one notable exception. The institutional hierarchy/professional rank was the only characteristic significantly associated with ED risk category (χ^2^(3) = 8.8, *p* = 0.032) among ballet dancers. Specifically, *corps de ballet* dancers had the highest proportion of clinically relevant ED symptomatology, accounting for 45.2% of the high-risk subgroup, whereas soloists had a noticeably lower proportion within their rank (12%). No statistically significant differences in ED symptomatology risk distribution were observed across age groups (*p* = 0.343), specific ballet theaters (*p* = 0.264), education or income levels (*p* = 0.582 and *p* = 0.984), seniority (*p* = 0.558), or marital status (*p* = 0.134). These findings suggest that ED symptomatology was broadly distributed across diverse demographic strata of ballet dancers, but varied according to professional rank within the theater hierarchy.

### 3.3. Psychosocial Working Conditions and Multivariable Associations with Eating Disorder Symptomatology

Subgroup bivariate analyses identified critical sociodemographic and institutional differences in perceived psychological stress at work. Standardized differences between groups are highlighted by the effect size (d) profile in Figure 5, and the absolute mean scores are presented in Table A1.

Lower monthly income range (600–1500 EUR) was associated with a large, statistically significant adverse effect on the ‘Role’ subscale (d = 0.8, 95% CI: 0.2; 1.2), reflecting greater role ambiguity and lower systemic autonomy than in the higher-income ballet dancers’ cohort (1501–5000 EUR). Lower income was also significantly related to elevated psychological distress regarding the HSE subscales, namely, ‘Managerial support’, ‘Control’, and ‘Change’ (d = 0.4, 95% CI: 0.1–0.8).

Lower-ranking dancers (*corps de ballet* and *coryphées*) reported systematically less favorable conditions across most HSE domains compared to soloists and principal dancers. More specifically, small-to-medium effect sizes were identified for ‘Role’ (d = 0.5, 95% CI: 0.1; 0.9), ‘Managerial support’ (d = 0.8, 95% CI: 0.4; 1.2), ‘Control’ (d = 0.5, 95% CI: 0.1; 0.9), ‘Change’ (d = 0.6, 95% CI: 0.2; 1.0), and ‘Peer support’ (d = 0.6, 95% CI: 0.2; 1.0) subscales of the HSE. The ‘Relationships’ subscale, which includes items related to workplace bullying and possible mobbing, also differed significantly by rank, with less favorable scores among lower-ranking ballet dancers (d = 0.4, 95% CI: 0.1; 0.8).

Marital status verified the ‘support-as-buffer’ framework. Single or non-married ballet dancers reported significantly lower job control (d = 0.6, 95% CI: 0.2; 0.9), lower peer support (d = 0.5, 95% CI: 0.1; 0.9), and more fractured interpersonal relationships (d = 0.4, 95% CI: 0.1; 0.8) than their married counterparts.

In the next stage of analysis, linear regression analyses of the various independent variables were performed to assess the predictive values of occupational stress for ED symptomatology in a cohort of ballet dancers (Figure 6).

In the linear regression models, the HSE core subscales served as independent variables, while the total EAT-26 score and the scores of its distinct subscales, Factor D, Factor B, and Factor O, were assigned as dependent variables. All linear regression models were adjusted for the potential confounding sociodemographic and institutional factors, including marital status, monthly income, and professional rank.

The linear regression models revealed that interpersonal and social dynamics within the company were the most consistent correlates of ED risk. In particular, the HSE ‘Relationships’ subscale demonstrated the strongest association across the examined EAT-26 outcomes (Table 3).

Higher friction and poorer interpersonal relationships were significantly associated with higher total EAT-26 scores (β 8.2, 95% CI: 3.8; 12.7, *p* < 0.001), accounting for 25% of the total variance (R^2^ = 0.25). Similarly, ‘Peer support’ was also significantly associated with total EAT-26 scores (β 6.4, 95% CI: 2.7; 10.1, *p* = 0.001, R^2^ = 0.22).

For the specific eating attitudes profiles, both ‘Relationships’ and ‘Peer support’ maintained their definitive predictive value, thus remaining among the strongest correlates. For Factor D, ‘Peer support’ (β 3.6, 95% CI: 1.5; 5.7, *p* = 0.001, R^2^ = 0.25) and ‘Relationships’ (β 4.1, 95% CI: 1.5; 6.6, *p* = 0.022, R^2^ = 0.23) explained the highest proportions of variance. By analogy, for Factor B, ‘Relationships’ was also significantly associated with the score (β 1.5, 95% CI: 0.5; 2.4, *p* = 0.002, R^2^ = 0.25).

For Factor O, identified as the dominant eating pattern in nearly half of the ballet dancers, ‘Relationships’ showed the strongest association (β 2.2, 95% CI: 0.9; 3.6, *p* = 0.001, R^2^ = 0.25). While other HSE occupational subscales, namely, ‘Control’, ‘Managerial support’, and ‘Peer support’, also showed statistically significant associations with Factor O, these models explained less variance.

Considering ‘Peer support’ and ‘Relationships’ showed the strongest and most consistent associations with the total EAT-26 scale and specific subscales, these HSE domains were selected as the primary target independent variables for subsequent granular item-level analysis to identify potential environmental factors associated with the expression of ED symptoms (Figure 7).

In the final linear regression model, all individual items from the HSE ‘Peer support’ and ‘Relationships’ subscales were entered simultaneously as independent variables with the total EAT-26 score as the dependent variable. As a result, the item-level analysis yielded highly targeted and precise outcomes. General social climate indicators within the company, including ‘Assistance from coworkers’ (β = 0.5, 95% CI: −2.2; 3.3, *p* = 0.692), ‘Positive peer support’ (β 0.4, 95% CI: −3.1; 4.0, *p* = 0.831), ‘Occupational respect’ (β 0.6, 95% CI: −3.3; 4.5, *p* = 0.749), and ‘Sounding board’ (β 1.6, 95% CI: −2.0; 5.1, *p* = 0.381) were not significantly associated with EAT-26 scores and were discarded as active potential risk factors for ED symptomatology development. Other items related to the structural workplace strain and difficult general relationships were also non-significant.

In this context, the linear regression model identified that adverse interpersonal behaviors were the sole significant variables associated with elevated ED risk screening scores. Specifically, both self-reported workplace bullying (β 3.5, 95% CI: 0.1; 7.1, *p* = 0.049) and possible mobbing (β 4.4, 95% CI: 0.1; 8.7, *p* = 0.045) were the only items significantly associated with higher ED symptomatology and had the strongest regression weights in the entire regression model.

These findings indicated that ED symptomatology within the ballet cohort was associated not simply with a generally unsupportive work environment or challenging professional relationships, but more specifically with exposure to interpersonal strain and adverse organizational dynamics, particularly self-reported bullying and possible mobbing. These potential risk factors were identified as key statistical correlates within the institutional hierarchy.

## 4. Discussion

### 4.1. Proportion and Behavioral Profiles of Eating Disorder Symptomatology

The principal finding of this study revealed that professional female ballet dancers represented a particularly vulnerable high-risk group for the expression of clinically relevant ED symptomatology compared with the high-risk student cohort. Within the paradigm of performance nutrition and clinical dietetics, in the ballet cohort, 35.9% confirmed that the professional ballet environment may act as a potent, institutional correlate for disordered eating behaviors. Our study results matched the epidemiological range reported in previous reviews of global dance populations by Arcelus et al. [13] and Nordin-Bates et al. [58].

Although Bulimia and Food Preoccupation (EAT-26 Factor B) remained predominant within the student cohort, the dietary behavior of ballet dancers was primarily characterized by severe Oral Control (EAT-26 Factor O). This selective expression of Factor O suggests that the ballet-specific ED profile may be characterized less by loss-of-control or binge-eating mechanisms and more by avoidant or restrictive eating patterns [59]. Such restrictive eating can contribute to elevated overall ED scores in dancers far above general population levels [13,60], and is associated with a high proportion of atypical ED profiles reported in elite performance subcultures [61].

### 4.2. Sociodemographic, Institutional Factors, and the Eating Disorder Risk Spectrum

Beyond the primary regression model analyses, subgroup bivariate comparisons revealed critical sociodemographic and institutional disparities in perceived psychological occupational stress in a sample of ballet dancers. A strong hierarchy-dependent gradient was identified: lower-rank ballet dancers (*corps de ballet* and *coryphées*) reported significantly higher psychological stress across most HSE subscales, including lower control, poorer managerial support, and strained interpersonal relationships, than higher-rank ballet dancers, namely, soloists and principal dancers. The final phase of the data analysis revealed that higher ED symptoms were significantly associated only with self-reported bullying and possible mobbing within the entire HSE ‘Relationships’ subscale. These findings suggest that exposure to the risk of workplace bullying and potential mobbing is not only unevenly distributed, but is also highly concentrated in lower positions within the institutional hierarchy, where job insecurity and power imbalances are most pronounced.

In an international context, this hierarchy-related distress is consistent with emerging research on sociopsychological audits of elite classical dance companies, describing classical dance institutions as highly stratified, structurally inflexible environments [13,15,62]. Junior ballet dancers may be disproportionately exposed to adverse peer dynamics or institutional bullying [30]. From a nutritional epidemiology perspective, such structural distress can act as a primary environmental factor for the tracking of restrictive eating patterns [13,14]. This concern is reinforced by evidence that severe energy deficiency and disordered eating among professional dancers may occur in cultures where lower-ranking dancers feel compelled to internalize severe dietary restriction as a mandatory condition for career progression and group acceptance [13,63].

Lower income (600–1500 EUR vs. 1501–5000 EUR per month) and marital status were also associated with differences in HSE scores. Lower-income ballet dancers reported less favorable ‘Role’, ‘Managerial support’, ‘Control’, and ‘Change’ scores, suggesting greater role ambiguity, lower autonomy, less managerial support, and higher psychological occupational stress related to workplace changes. In addition, ballet dancers who were single or in non-marital relationships reported significantly lower job control, lower peer support, and more fractured interpersonal relationships compared to their married counterparts.

Under these circumstances, the combination of low income level and professional instability may increase vulnerability to role conflict and reduced psychological safety in the creative sector [15,31,64]. Ballet dancers are also a population susceptible to compulsive work habits [65], operating within lower economic limits, likely leading to systemic insecurity and chronic psychological stress, insufficient nutritional intake, and injury risk [66]. The marital status findings verify the ‘support-as-buffer’ hypothesis [67]. Thus, in an elite industry with hyper-competitive professional peer networks, an external formalized domestic partnership may provide vital emotional stabilization [68]. Conversely, the study indicates that ballet dancers who are theoretically limited in these external resources of support may rely entirely on the internal theater environment, potentially co-occurring with elevated rates of body checking, psychological exhaustion, and total estrangement [69].

### 4.3. Eating Inhibition as an Exploratory Somatic Framework for Reclaiming Autonomy

Given the cross-sectional design of this study, it did not directly measure neural activity. However, the identified association between perceived occupational stress, in terms of self-reported workplace bullying and possible mobbing, and the expression of clinically relevant ED symptomatology among ballet dancers provided an empirical baseline consistent with previous neurobiological literature. In order to assist future exploratory research, we projected these statistical associations onto a hypothetical socio-neuro-behavioral framework (Figure 8).

Within this conceptual framework and theoretical illustration, self-reported exposure to workplace bullying, possible mobbing, and diminished peer support captured by the HSE scale were hypothesized as potential correlates of neuroendocrine activation.

The pronounced oral control and food avoidance observed in ballet dancers may therefore be interpreted not only as isolated behavioral symptoms but also, hypothetically, as a compensatory top-down cognitive mechanism managed by prefrontal networks within the dorsolateral prefrontal cortex and medial prefrontal cortex [36] that may modulate amygdala-mediated anxiety [37]. The developed model conceptualized how this subcortical distress may map onto theoretical cortical models. Institutional vulnerability, including lower professional rank and income, may statistically overlap with greater exposure to adverse workplace relationships characterized by severe interpersonal friction, especially self-reported bullying, and possible mobbing (in terms of the HSE ‘Relationships’ scale).

At a theoretical subcortical level, chronic threat perception is hypothesized in the current literature to promote neuroendocrine activation which may involve amygdala hyperactivation, sustained cortisol secretion, and chronic hypothalamic–pituitary–adrenal (HPA) axis dysregulation [34,35]. However, it must be explicitly emphasized that these neural pathways and mechanisms were not measured or demonstrated by the present sample data; thus, the assigned standardized regional values in Figure 8 represent speculative weights conceptualized from theoretical frameworks to guide future hypothesis testing.

Clinically relevant ED symptomatology (EAT-26 ≥ 20) was observed in 35.9% of the ballet cohort. In ballet dancers, the restrictive profile identified in this study was heavily polarized toward oral control (EAT-26: Factor O, 49.1% of participants), which was characterized by potential dietary restraint [14]. The qualitative shift from the student bulimic profile, characterized by Factor B, to the ballet dancer restrictive profile, characterized by Factor O, may indicate that restrictive eating in dancers is more closely associated with behavioral characteristics in a rigid workplace environment.

The findings of our study support the hypothesis that the pronounced oral control among ballet dancers may function as a potential behavioral marker of a theoretical coping response, although this interpretation remains strictly speculative. Neuroimaging studies suggest that rigid behavioral restriction may temporarily reduce amygdala-driven anxiety, while providing a pseudo-sense of environmental autonomy. Over time, the combination of chronic hypercortisolemia and starvation may impair insular cortex functionality, disrupting interoceptive awareness of hunger and satiety, potentially reinforcing restrictive ED patterns related to self-reported systemic occupational strain [70]. Alternative explanations related to body image concerns, perfectionism, professional weight expectations, dietary practices, social comparison, and appearance evaluation should also be acknowledged as potential confounding variables [14].

According to the results obtained from this study, among lower-ranking [62] *corps de ballet* and *coryphées* and economically vulnerable ballet dancers, these processes may be further compounded by limited occupational control and managerial support. Accordingly, severe oral control and voluntary food avoidance could be considered not only in relation to aesthetic pressures or individual psychological characteristics, but also as potential behavioral patterns tracking with chronic occupational strain across ballet dancer populations [71]. These interconnected observations lay a conceptual baseline, providing the logical framework to establish specific inductive hypotheses and limitations requiring prospective verification in future investigations.

### 4.4. Study Limitations, Inductive Hypotheses, Future Directions, and Practical Implications

Several limitations should be considered when interpreting these findings. First, depending on the cross-sectional design of the study, causal associations between occupational stress, self-reported workplace bullying, and ED symptomatology cannot be definitively established. Although the multiple linear regression models identified significant associations, bidirectionality is possible; participants with higher expression of ED symptoms may also perceive workplace dynamics or interpersonal relationships differently. Longitudinal cohort studies are therefore needed to examine temporal relationships.

Second, workplace-related stress was assessed using the HSE Indicator Tool exclusively within the sample of ballet dancers, whereas the comparison group of students was excluded from this stage. This decision was based on the fact that higher education students were not engaged in formal professional employment; thus, their workplace health environment was methodologically incompatible. Consequently, perceived psychological stress could not be compared directly between the two cohorts. Future research could use general or academic stress instruments alongside occupational measures to enable such comparisons.

Third, data collection relied entirely on self-report questionnaires (EAT-26 and HSE Indicator Tool) without verifying findings through gold-standard diagnostic clinical interviews, introducing the possibility of social or retrospective recall bias. This may be particularly relevant in competitive ballet environments where disclosing mental health concerns or workplace bullying could be perceived as professionally disadvantageous. Additionally, this study lacked direct measurements of several theoretically important variables known to affect eating behavior, such as baseline body dissatisfaction and perfectionism, creating a risk for residual confounding.

Fourth, given the relatively modest ballet subsample (*n* = 117) and the multiple exploratory analyses conducted across parallel HSE dimensions, individual items, and EAT-26 subscales, the statistical models face an inflated risk of Type I errors. Consequently, the findings—particularly marginal values—must be interpreted with strict scientific caution rather than as definitive evidence of primary environmental correlates, especially given that exploratory paths for self-reported bullying and potential mobbing were only marginally below the conventional significance threshold (*p* = 0.049 and *p* = 0.045).

Finally, although the sample of ballet dancers (*n* = 117) was relatively large compared with samples in previous studies in this field, participants were recruited from a limited number of institutions. The findings may therefore not be fully generalizable to alternative dance disciplines, freelance dancers, or different geographical and cultural settings in which management styles in ballet and social support systems may differ.

Depending on the empirical data and the distinct behavioral configurations identified in the present study cohort, we propose three inductive hypotheses for future prospective verification:

The Behavioral Divergence Hypothesis: Psychological stress associated with structural, hierarchical strain is linked with a restrictive ED pattern (Factor O), whereas academic psychological stress correlates primarily with a predominantly emotional or bulimic ED profile (Factor B).

The Autonomy Proxy Hypothesis: In elite aesthetic environments, the expression of Factor O (oral control) is closely associated with the perceived loss of organizational autonomy rather than exclusively with the desire for thinness.

The Hierarchy-Driven Vulnerability Hypothesis: Lower institutional rank (*corps de ballet*/*coryphées*) represents a core psychosocial vulnerability that may increase the likelihood of progression from sub-clinical eating distress tracking into clinical risk.

Future research should extend these findings in two ways. First, longitudinal cohort studies should follow young ballerinas as they transition from pre-professional training to professional corporate structures. This future direction would allow researchers to determine whether changes in exposure to workplace bullying and possible mobbing and changes in institutional rank precede changes in Factor O (oral control) symptomatology. Second, the proposed neurobiological framework should be empirically tested by combining ED screening with functional neuroimaging. For example, fMRI studies should compare ballet dancers reporting high vs. low exposure to workplace bullying or possible mobbing while presenting body image and food stimuli.

The findings of this study also suggest potential practical implications for nutritional science, public health, ballet management, and dance medicine, which should be framed strictly as hypotheses requiring prospective evaluation: (1) nutritional education or dietetic counseling alone may be insufficient when restrictive or bulimic eating behaviors occur as behavioral responses to chronic workplace strain within a rigid institutional hierarchy, traditional nutritional care in ballet should therefore be integrated with appropriate occupational and psychological support; (2) given that perceived relational strain correlates with higher scores for ED symptomatology, psychologically safe working environments should be considered an important component for protecting ballet dancers’ nutritional and metabolic health; (3) organizational health monitoring may benefit from validated tools such as the HSE Indicator Tool to assess psychosocial working conditions.

## 5. Conclusions

This study identified an exploratory association between self-reported workplace bullying and possible mobbing as occupational stressors and greater ED symptomatology among female ballet dancers. These findings suggest that perceived psychological stress within the workplace may be a significant correlate of ED risk within professional ballet.

Psychometric profiling of female ballet dancers indicated a distinct ballet-related pattern characterized by pronounced oral control (EAT-26 Factor O), consistent with restrictive eating patterns and potential avoidant behaviors. Within an exploratory framework, this pattern may represent a behavioral coping response to maintain internal stability in rigid theater environments and to deal with the perceived occupational psychological stress. Female ballet dancers with lower professional status and income levels, alongside a lack of protective social support, demonstrated higher vulnerability to occupational stressors. Therefore, eating disorder-related risks in ballet should be addressed not merely as individual pathologies, but as multi-level outcomes interacting with rigid institutional hierarchies and theoretical neurobiological stress models.

From a nutritional science and public health perspective, the findings imply that nutritional interventions addressing EDs and low energy availability in dancers should not rely on nutritional counseling alone. Multi-level approaches that also address workplace mental health, interpersonal safety, and organizational culture may better support healthy eating patterns and nutritional well-being among female ballet dancers, though these prospective strategies require future validation through longitudinal or intervention-based evidence.

## Figures and Tables

**Figure 1:**
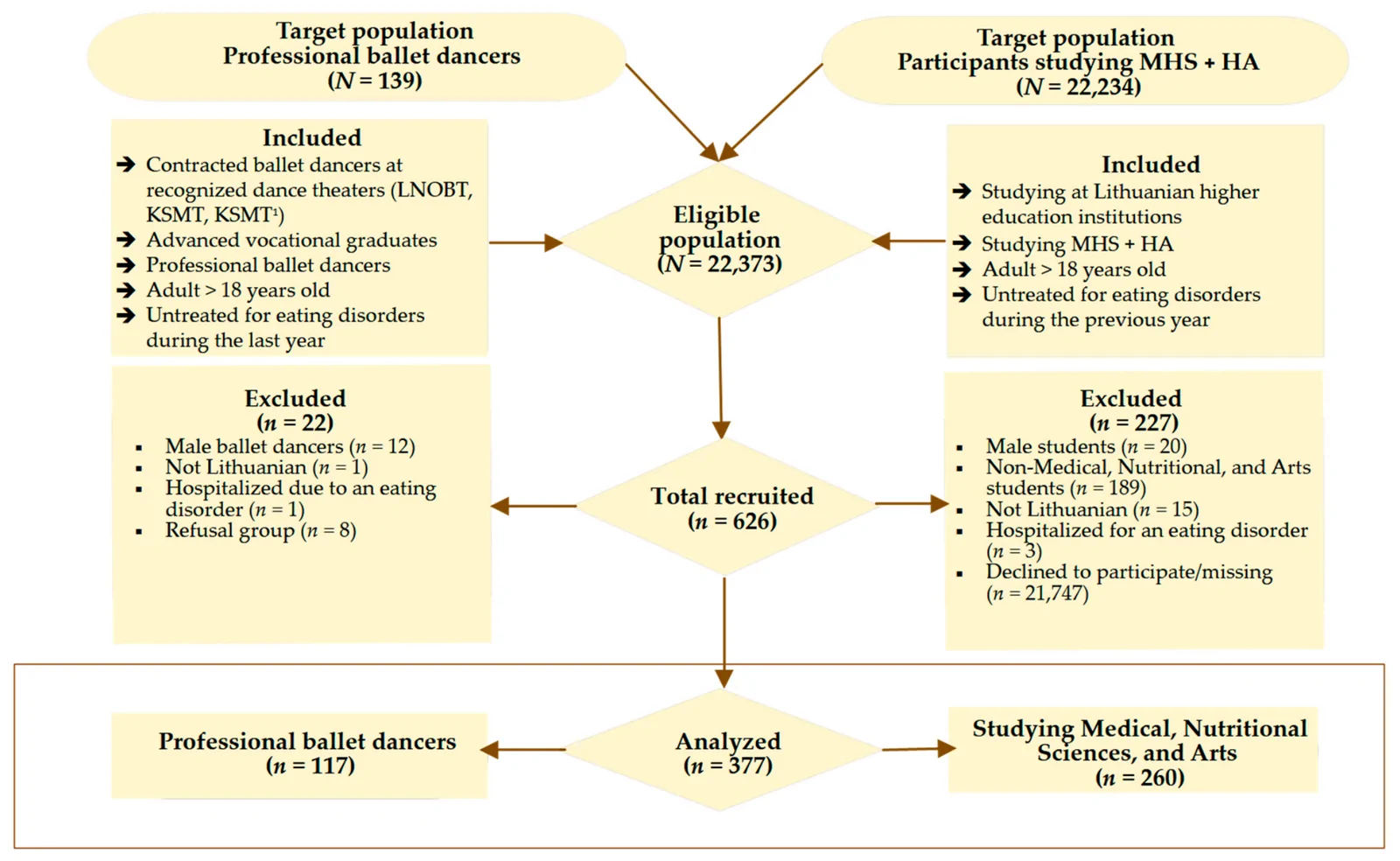
Flowchart of participant recruitment and screening illustrating the inclusion and exclusion criteria used to derive the final analyzed cohorts of professional female ballet dancers (*n* = 117) and higher education students (*n* = 260). HA—Humanities and Arts; KSMT—Kaunas State Musical Theatre; KSMT^1^—Klaipėda State Musical Theatre; LNOBT—Lithuanian National Opera and Ballet Theatre; MHS—Medical and Health Sciences (including Medical and Nutritional sub-disciplines).

**Figure 2:**
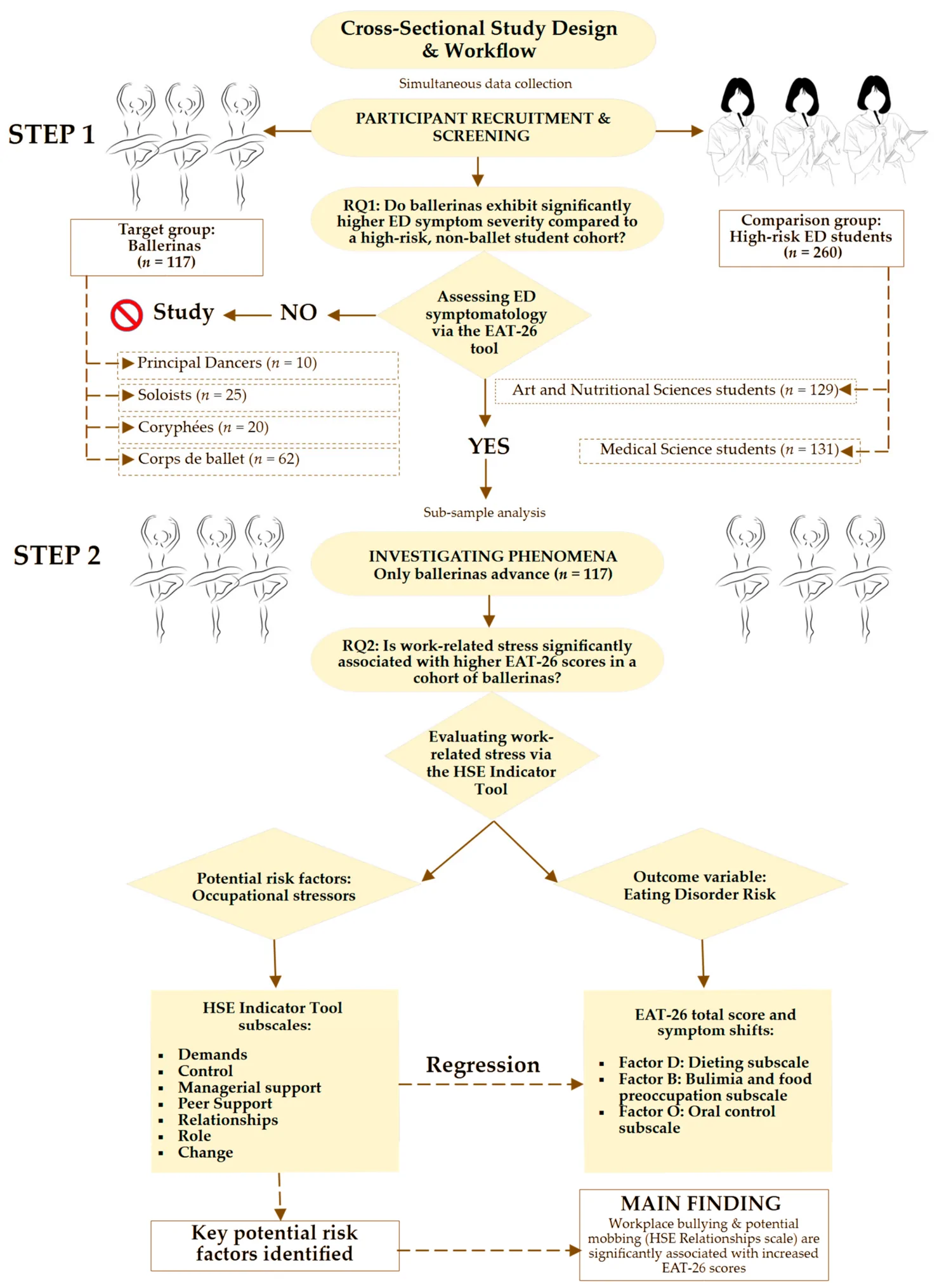
Flowchart of the cross-sectional study design and methodological workflow. Step 1 illustrates participant recruitment, screening, and baseline comparison of ED symptomatology (RQ1) between ballet dancers (*n* = 117) and the student cohort (*n* = 260). Step 2 shows the subsample analysis focusing exclusively on the ballet cohort (RQ2), which evaluated potential occupational risk factors using the HSE Indicator Tool and multivariable linear regression. EAT-26—Eating Attitudes Test-26; ED—eating disorder; HSE—Health and Safety Executive; RQ—research question.

**Figure 3:**
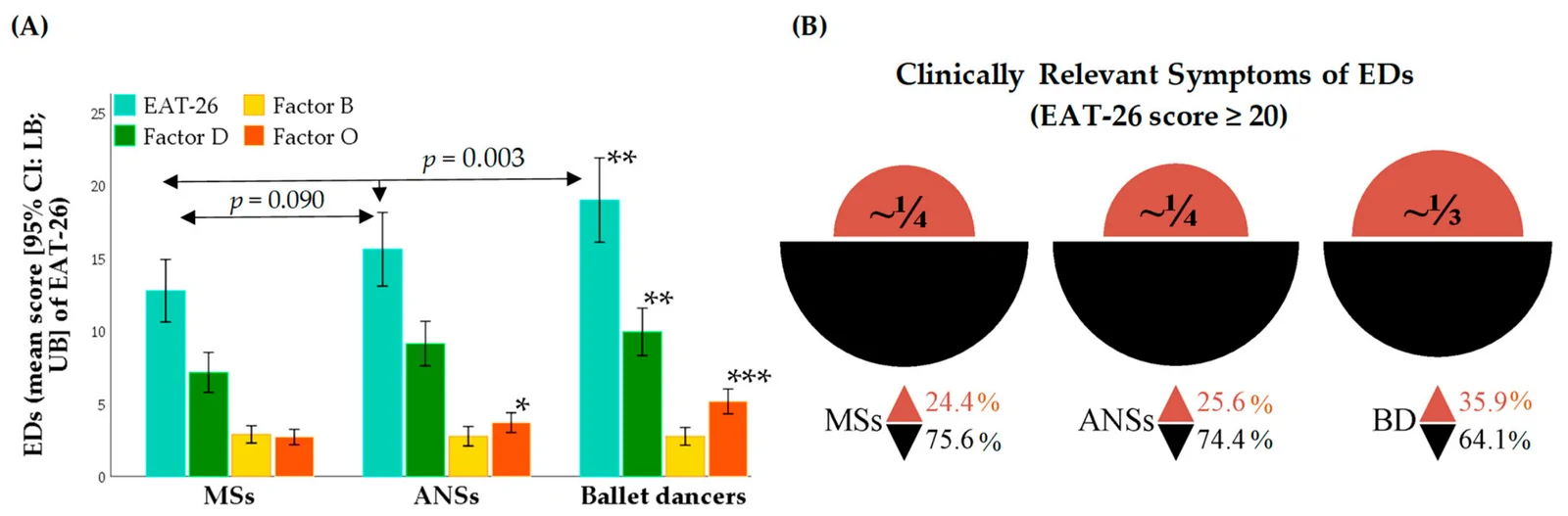
Comparison of eating disorder risk and eating attitudes across cohorts: (**A**) Mean EAT-26 global scores and subscale scores (Factors D, B, and O) among MSs, ANSs, and ballet dancers; (**B**) Proportion of participants with clinically relevant eating disorder symptoms (EAT-26 score ≥ 20) across study groups. ANSs—Arts and Nutritional Sciences students; BD—ballet dancers; CI—confidence interval; EAT-26—Eating Attitudes Test-26; ED—eating disorder; LB—lower bound; MSs—Medical Science students; UB—upper bound. *—*p*-value ≤ 0.05; **—*p*-value ≤ 0.01; ***—*p*-value ≤ 0.001.

**Figure 4:**
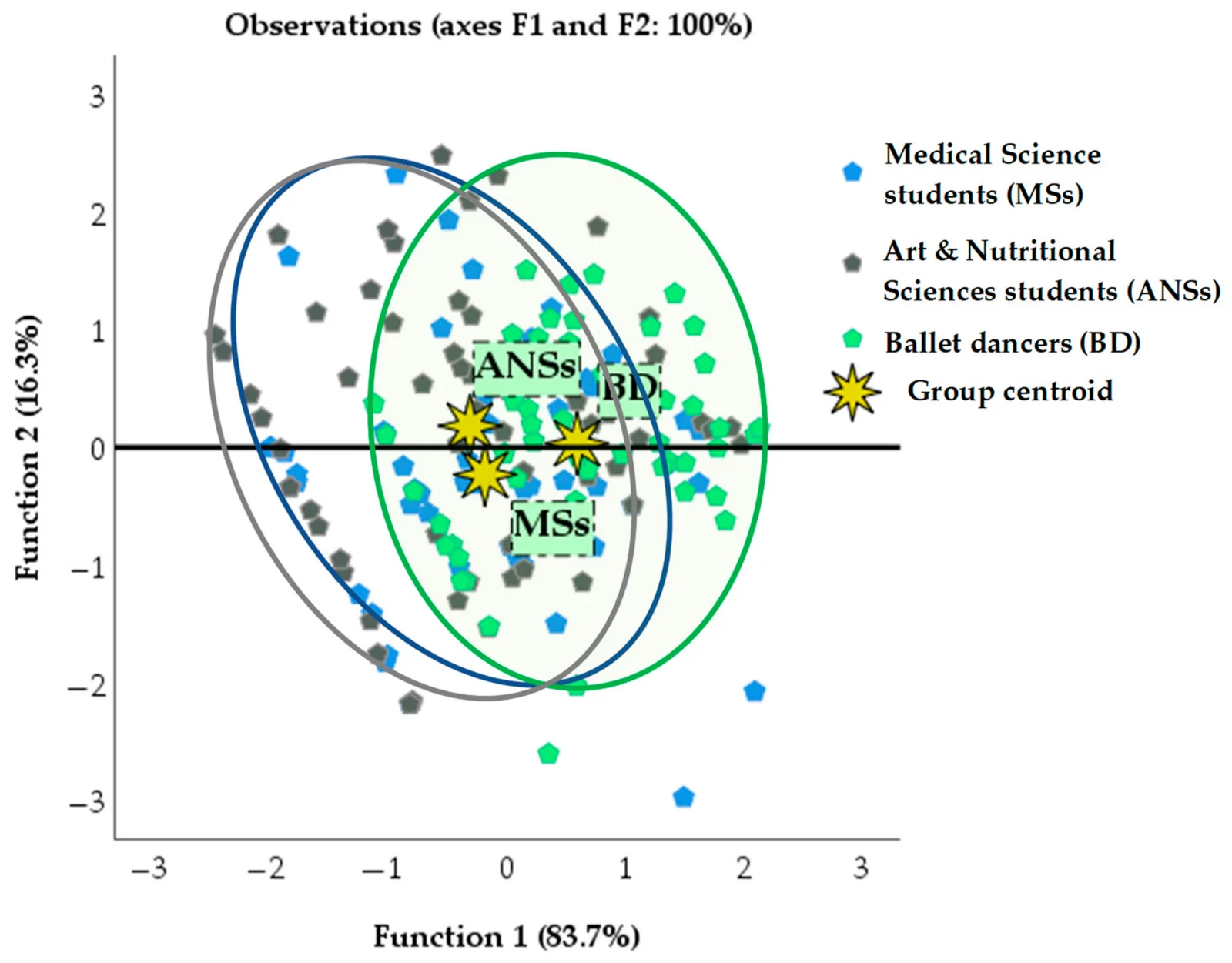
Discriminant function scatter plot with confidence ellipses showing the statistical distribution of eating behavior profiles based on the EAT-26 subscale dimensions across the three studied cohorts. Function 1 (83.7% of variance) and Function 2 (16.3% of variance) were the canonical discriminant functions derived from the stepwise linear discriminant analysis (LDA). Yellow stars indicate group centroids, while ellipses represent the 95% confidence intervals for each respective group. ANSs—Arts and Nutritional Sciences students; BD—ballet dancers; EAT-26—Eating Attitudes Test-26; MSs—Medical Science students.

**Figure 5:**
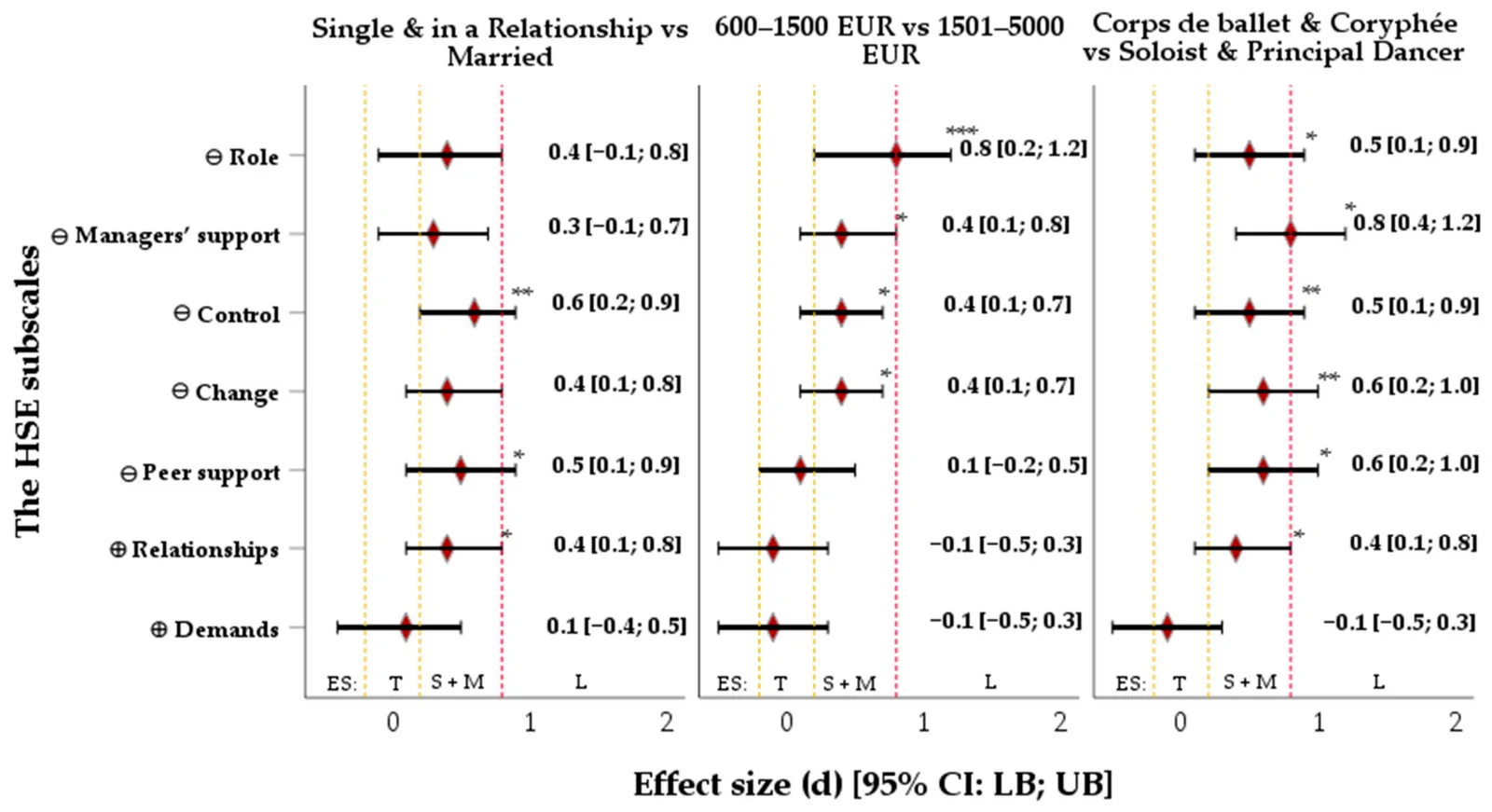
Forest plot of effect sizes (Cohen’s d) with 95% confidence intervals comparing perceived psychosocial workplace stressors (HSE core subscales) across sociodemographic and institutional subgroups of ballet dancers. The ⊖ and ⊕ symbols adjacent to the titles of each HSE subscale indicate the coding direction of the corresponding questionnaire items. CI—confidence interval; ES—effect size; HSE—Health and Safety Executive; L—large effect; LB—lower bound; S + M—small to medium effect; T—trivial effect; UB—upper bound. * *p*-value ≤ 0.05; ** *p*-value ≤ 0.01; *** *p*-value ≤ 0.001.

**Figure 6:**
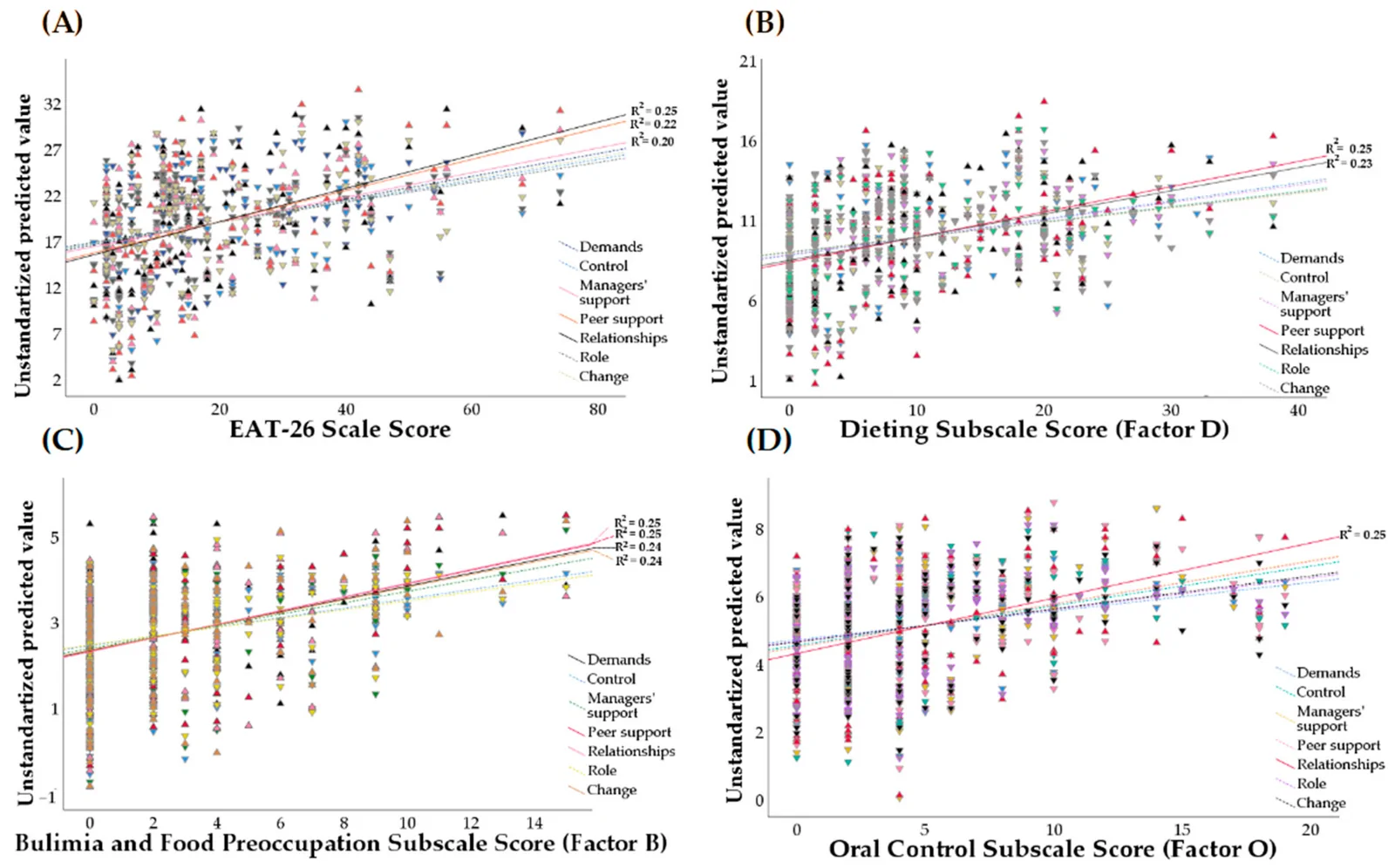
(**A**–**D**). Multivariable linear regression trajectories relating HSE core occupational stress dimensions to total EAT-26 and subscale scores. The *Y*-axis represents unstandardized predicted values generated from independent multivariable models in which each respective HSE subscale was the primary independent variable and institutional rank, monthly income, and marital status were covariates. Solid lines indicate statistically significant linear associations and display the corresponding adjusted coefficient of determination (R^2^); dashed lines represent statistically non-significant regression pathways. (**A**) Association between HSE domains and total EAT-26 scores; (**B**) Relationship between HSE domains and Dieting subscale (Factor D) scores; (**C**) Association between HSE domains and Bulimia and Food Preoccupation subscale (Factor B) scores; (**D**) Relationship between HSE domains and Oral Control subscale (Factor O) scores. EAT-26—Eating Attitudes Test-26; HSE—Health and Safety Executive. A more detailed description can be found in Table 3.

**Figure 7:**
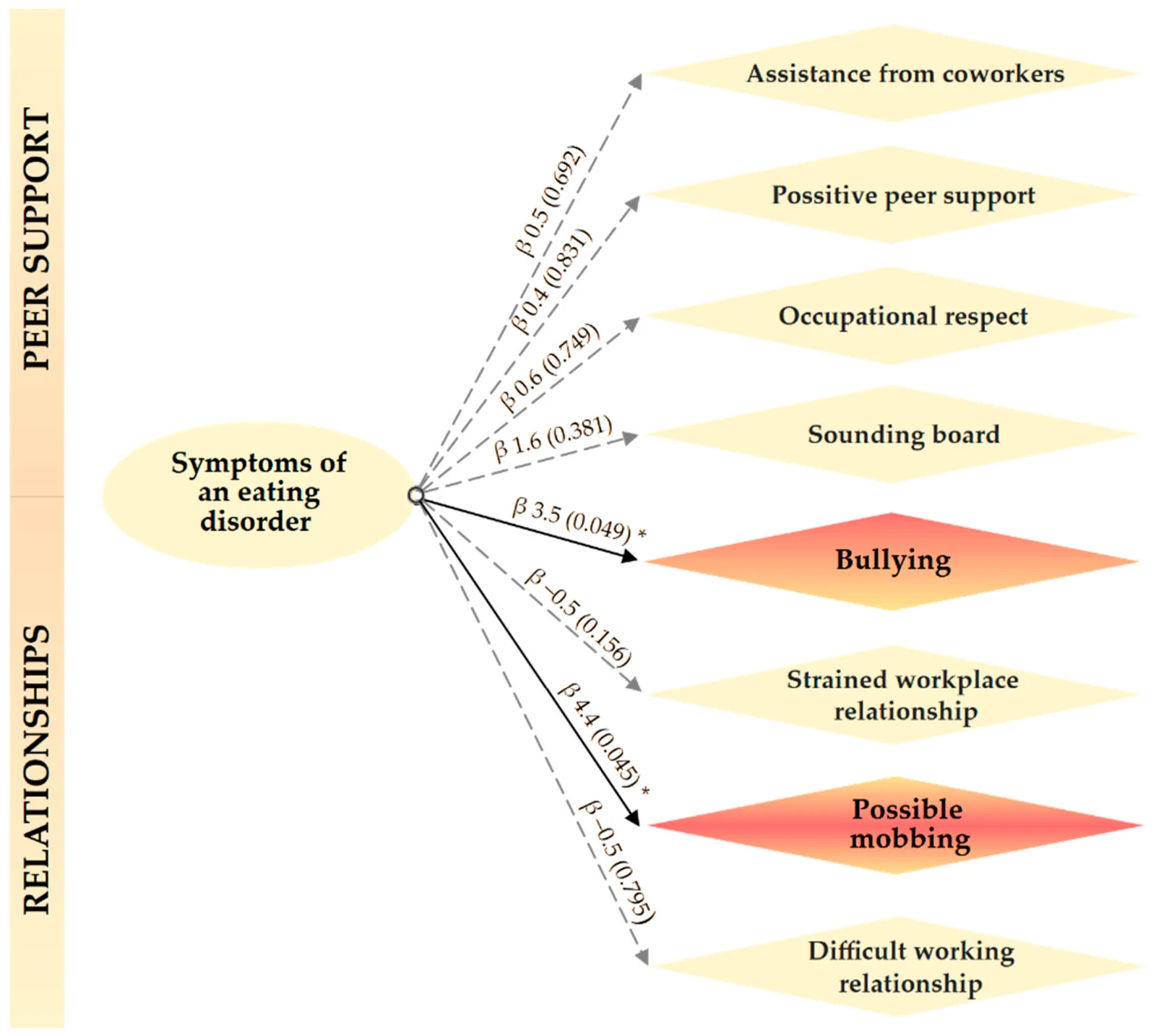
Item-level multivariable regression model relating ED symptomatology to individual items from the HSE ‘Peer support’ and ‘Relationships’ subscales. Model fitting criteria: F (11, 104) = 5.1, *p* < 0.001, R^2^ = 0.31. Solid black lines represent statistically significant associations between variables. Dashed gray lines indicate statistically non-significant associations. β—standardized coefficient; HSE—Health and Safety Executive; *p*—*p*-value; R^2^—coefficient of determination; * *p*-value ≤ 0.05.

**Figure 8:**
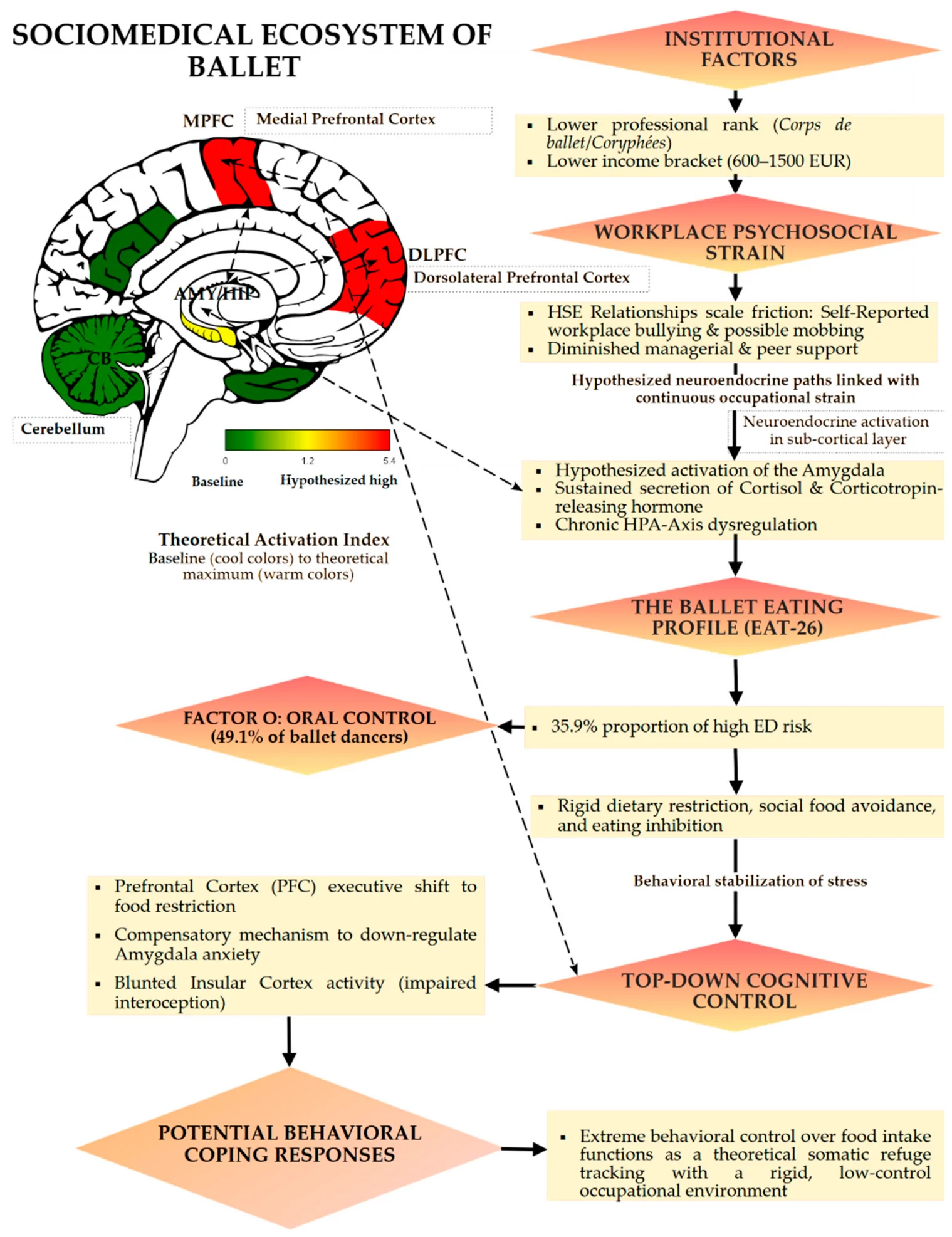
Hypothetical socio-neuro-behavioral model conceptualizing exploratory associations in the ballet eating profile. The brain visualization and color scale represent a purely theoretical mapping of assigned regional weights conceptualized from theoretical frameworks—ranging from baseline (cool colors) to a hypothesized high expression or theoretical maximum (warm colors)—and do not represent empirical neural or neuroendocrine activity measured within the present sample. AMY—amygdala; DLPFC—dorsolateral prefrontal cortex; EAT-26—Eating Attitudes Test-26; HPA—hypothalamic–pituitary–adrenal; MPFC—medial prefrontal cortex.

**Table 1:** Stratification of ballet dancers by eating disorder symptomatology levels across sociodemographic and occupational characteristics (*n* = 117).

Variables	Total		Eating Disorder Symptoms				χ^2^ (*p*)	OR [95% CI: LB; UB]
			ED Risk (−) EAT-26 Score ≤ 19		ED Risk (+) EAT-26 Score ≥ 20			
	*n*	%	*n*	%	*n*	%		
Age groups								
20- to 22-year-olds	60	51.3	36	60.0	24	40.0	0.9 (0.343)	1.4 [0.7; 3.1]
23- to 45-year-olds	57	48.7	39	68.4	18	31.6		1
Ballet theater								
LNOBT	76	65.0	46	60.5	30	39.5	2.7 (0.264)	2.6 [0.8; 8.6]
KSMT	21	17.9	13	61.9	8	38.1		2.5 [0.6; 10.0]
KSMT^1^	20	17.1	16	80.0	4	20.0		1
Ballet dancer rank								
Principal Dancer	10	8.5	7	70.0	3	30.0	8.8 (0.032)	0.5 [0.1; 2.2]
Soloist	25	21.4	22	88.0	3	12.0		0.2 [0.1; 0.6]
*Coryphée*	20	17.1	12	60.0	8	40.0		0.8 [0.3; 2.2]
*Corps de ballet*	62	53.0	34	54.8	28	45.2		1
Education levels								
College	68	58.1	45	66.2	23	33.8	0.3 (0.582)	1
University	49	41.9	30	61.2	19	38.8		1.2 [0.6; 2.7]
Income status								
600–1500 EUR	67	57.3	43	64.2	24	35.8	0.1 (0.984)	1
1501–5000 EUR	50	42.7	32	64.0	18	36.0		1.1 [0.5; 2.2]
Seniority								
1–7 years	66	56.4	40	60.6	26	39.4	0.8 (0.370)	1.4 [0.7; 3.1]
8–20 years	51	43.6	35	68.6	16	31.4		1
Marital status								
Single or in a relationship	82	70.1	49	59.8	33	40.2	2.3 (0.134)	1.9 [0.8; 4.7]
Married	35	29.9	26	74.3	9	25.7		1

CI—confidence interval; EAT-26—Eating Attitudes Test-26; ED—eating disorder; χ^2^—the chi-squared test; KSMT—Kaunas State Musical Theatre; KSMT^1^—Klaipėda State Musical Theatre; LNOBT—the Lithuanian National Opera and Ballet Theatre; LB—lower bound; OR—odds ratio; UB—upper bound; *p*—*p*-value.

**Table 2:** Descriptive statistics and comparative analysis of EAT-26 total and subscale scores (Factors D, B, and O) between female ballet dancers and student comparison subgroups.

Eating Disorder Symptoms	Ballet Dancers (*n* = 117)	Comparison Group			d [95% CI: LB; UB]	*p*
		ANS (*n* = 129)	MS (*n* = 131)	ANS + MS (*n* = 260)		
	Mean [95% CI: LB; UB]					
EAT-26 score	19.0 [16.1; 21.9]	15.6 [13.1; 18.1]	12.8 [10.6; 14.9]	14.2 [12.5; 15.8]	0.3 [0.1; 0.6]	0.003
Factor D score	9.9 [8.3; 11.6]	9.1 [7.6; 10.7]	7.2 [5.8; 8.5]	8.1 [7.1; 9.2]	0.3 [0.1; 0.4]	0.049
Factor B score	2.8 [2.2; 3.4]	2.8 [2.1; 3.4]	2.9 [2.3; 3.5]	2.8 [2.4; 3.3]	−0.01 [−0.2; 0.2]	0.859
Factor O score	5.2 [4.3; 6.1]	1.6 [3.0; 4.4]	2.7 [2.2; 3.3]	3.2 [2.8; 3.6]	0.5 [0.3; 0.7]	<0.001

Data are expressed as mean [95% CI: LB; UB]. d—Cohen’s d effect size for the comparison between ballet dancers and the pooled student group (ANS + MS). The ‘ANS + MS’ column represents the pooled student comparison group. ANSs—Arts and Nutritional Sciences students; BD—ballet dancers; EAT-26—Eating Attitudes Test-26; Factor B—Bulimia and Food Preoccupation subscale; Factor D—Dieting subscale; Factor O—Oral Control subscale; LB—lower bound; MSs—Medical Science students; *p*—*p*-value; UB—upper bound.

**Table 3:** Associations between psychosocial working conditions (HSE Indicator Tool) and eating attitudes (EAT-26) scale and subscale scores (multivariable regression models).

Dependent Variables	The HSE Core Subscales as Independent Variables						
	Demands	Control	Managerial Support	Peer Support	Relationships	Role	Change
EAT-26 scale							
β (95% CI [LB; UB])	6.3 [0.9; 11.8]	4.1 [0.1; 8.2]	5.3 [1.2; 9.3]	6.4 [2.7; 10.1]	8.2 [3.8; 12.7]	4.4 [−0.5; 9.2]	3.7 [0.2; 7.1]
*p*	0.023	0.050	0.011	0.001	<0.001	0.075	0.036
F (4, 112)	4.0	3.6	4.3	5.7	6.2	3.4	3.8
R^2^	0.12	0.11	0.20	0.22	0.25	0.11	0.12
Factor D subscale							
β (95% CI [LB; UB])	3.6 [0.5; 6.7]	1.9 [−0.4; 4.3]	2.6 [0.3; 4.9]	3.6 [1.5; 5.7]	4.1 [1.5; 6.6]	2.5 [−0.3; 5.2]	1.7 [−0.3; 3.7]
*p*	0.022	0.109	0.030	0.001	0.022	0.077	0.090
F (4, 112)	3.6	2.8	3.4	5.2	4.7	2.9	2.9
R^2^	0.11	0.10	0.11	0.25	0.23	0.10	0.10
Factor B subscale							
β (95% CI [LB; UB])	1.6 [0.5; 2.7]	0.7 [−0.2; 1.6]	1.0 [0.2; 1.9]	1.2 [0.4; 2.0]	1.5 [0.5; 2.4]	0.7 [−0.3; 1.7]	1.0 [0.3; 1.7]
*p*	0.005	0.109	0.017	0.003	0.002	0.179	0.006
F (4, 112)	5.0	3.5	4.3	5.3	5.4	3.2	5.0
R^2^	0.24	0.11	0.13	0.25	0.25	0.10	0.24
Factor O subscale							
β (95% CI [LB; UB])	0.6 [−0.1; 2.2]	1.3 [0.1; 2.5]	1.5 [0.3; 2.7]	1.4 [0.2; 2.5]	2.2 [0.9; 3.6]	0.9 [−0.5; 2.4]	0.7 [−0.3; 1.8]
*p*	0.476	0.034	0.015	0.017	0.001	0.207	0.150
F (4, 112)	2.6	3.7	4.1	4.1	5.5	2.9	3.0
R^2^	0.10	0.12	0.13	0.13	0.25	0.10	0.10

Linear regression models were adjusted for the ballet dancers’ institutional rank, monthly income, and marital status as covariates. β—unstandardized coefficient; CI—confidence interval; EAT-26—Eating Attitudes Test-26; F—F-statistic; Factor B—Bulimia and Food Preoccupation subscale; Factor D—Dieting subscale of EAT-26; Factor O—the Oral Control subscale of EAT-26; HSE—Health and Safety Executive; LB—lower bound; UB—upper bound; R^2^—coefficient of determination.

## Data Availability

The data presented in this study are available on request from the corresponding author unless sharing them would go against ethical considerations, such as study participants’ consent and privacy.

## Supplementary Materials

The following supporting information can be downloaded at: https://www.mdpi.com/article/10.3390/nu18193191/s1. Table S1: Internal consistency and item-level reliability analysis of the Eating Attitudes Test (EAT-26) scale and its subscales (Dieting, Bulimia and Food Preoccupation, and Oral Control) within the study sample (*n* = 377); Table S2: Internal consistency and item-level reliability analysis of the 35-item Health and Safety Executive (HSE) Indicator Tool and its seven core subscales within the professional ballet dancer cohort (*n* = 117).

## Abbreviations

The following abbreviations are used in this manuscript:

AMY	Amygdala
BD	Ballet dancers
CI	Confidence interval
DEAs	Disordered eating attitudes
DLPFC	Dorsolateral prefrontal cortex
EAT-26	Eating Attitudes Test-26
ED	Eating disorder
Factor B	Bulimia and Food Preoccupation subscale of EAT-26
Factor D	Dieting subscale of EAT-26
Factor O	Oral Control subscale of EAT-26
fMRI	Functional magnetic resonance imaging
HPA	Hypothalamic–pituitary–adrenal
HSE	Health and Safety Executive
LDA	Linear discriminant analysis
MHS	Medical and Health Sciences
MPFC	Medial prefrontal cortex
OR	Odds ratio
RQ	Research question

## Appendix A

**Table A1:** Distribution of the HSE core subscale scores among ballet dancers stratified by marital status, income, and professional rank (*n* = 117).

The HSE Core Subscales	Marital Status			Income Level		Ballet Dancer Ranks	
	Total	Single & in a Relationship (*n* = 82)	Married (*n* = 35)	600–1500 EUR (*n* = 67)	1501–5000 EUR (*n* = 50)	*Corps de Ballet* & *Coryphée* (*n* = 82)	Soloist & Principal Dancer (*n* = 35)
	Mean (95% CI [LB; UB])						
Demands	2.7 [2.6; 2.9]	2.8 [2.7; 2.9]	2.8 [2.6; 2.9]	2.7 [2.6; 2.9]	2.8 [2.6; 2.9]	2.7 [2.6; 2.8]	2.8 [2.6; 3.0]
Control	3.3 [3.1; 3.4]	3.4 [3.3; 3.5]	2.9 [2.7; 3.3]	3.4 [3.2; 3.5]	3.1 [2.9; 3.4]	3.4 [3.2; 3.5]	3.0 [2.7; 3.3]
Managerial support	2.8 [2.6; 2.9]	2.9 [2.7; 3.0]	2.6 [2.4; 2.9]	2.9 [2.7; 3.1]	2.6 [2.4; 2.8]	3.0 [2.8; 3.1]	2.4 [2.1; 2.7]
Peer support	2.6 [2.5; 2.8]	2.7 [2.6; 2.9]	2.4 [2.1; 2.6]	2.7 [2.5; 2.9]	2.6 [2.3; 2.8]	2.8 [2.6; 2.9]	2.3 [2.0; 2.6]
Relationships	2.6 [2.5; 2.7]	2.6 [2.5; 2.8]	2.4 [2.2; 2.6]	2.6 [2.4; 2.7]	2.6 [2.4; 2.8]	2.6 [2.5; 2.8]	2.4 [2.2; 2.6]
Role	1.9 [1.7; 2.0]	1.9 [1.8; 2.1]	1.7 [1.5; 1.9]	2.1 [1.9; 2.2]	1.6 [1.4; 1.7]	2.0 [1.8; 2.1]	1.7 [1.5; 1.8]
Change	2.8 [2.7; 3.0]	2.8 [2.7; 2.9]	2.6 [2.3; 2.9]	3.0 [2.8; 3.2]	2.7 [2.4; 2.9]	3.0 [2.8; 3.2]	2.5 [2.2; 2.8]

Data are presented as means with 95% CIs [LB; UB]. CI—confidence interval; HSE—Health and Safety Executive; LB—lower bound; UB—upper bound.

## References

[1] Galmiche M., Déchelotte P., Lambert G., Tavolacci M.P. (2019). Prevalence of eating disorders over the 2000–2018 period: A systematic literature review. Am. J. Clin. Nutr..

[2] American Psychiatric Association (2022). Diagnostic and Statistical Manual of Mental Disorders: Fifth Edition, Text Revision (DSM-5-TR).

[3] van Hoeken D., Hoek H.W. (2020). Review of the burden of eating disorders: Mortality, disability, costs, quality of life, and family burden. Curr. Opin. Psychiatry.

[4] Smink F.R., Van Hoeken D., Hoek H.W. (2012). Epidemiology of eating disorders: Incidence, prevalence and mortality rates. Curr. Psychiatry Rep..

[5] Culbert K.M., Racine S.E., Klump K.L. (2015). Research Review: What we have learned about the causes of eating disorders–A synthesis of sociocultural, psychological, and biological research. J. Child Psychol. Psychiatry.

[6] Ortiz A.M.L., Peters A.S., Webber K.T., Butler R.M., Fitterman-Harris H.F., Levinson C.A. (2026). Eating disorder symptoms and corresponding evidence-based treatments: A narrative review. J. Eat. Disord..

[7] Varela C., Hoyo Á., Tapia-Sanz M.E., Jimenez-Gonzalez A.I., Moral B.J., Rodriguez-Fernandez P., Vargas-Hernández Y., Ruiz-Sanchez L.J. (2023). An update on the underlying risk factors of eating disorders onset during adolescence: A systematic review. Front. Psychol..

[8] Gagné-Pomerleau É., Bédard A., Savard C., Bolduc M., Gagnon-Girouard M.P., Lemieux S., Bégin C. (2026). Disordered eating profiles in the general adult population: A scoping review. Appet. Disord. Eat..

[9] López-Gil J.F., García-Hermoso A., Smith L., Firth J., Trott M., Mesas A.E., Jiménez-López E., Gutiérrez-Espinoza H., Tárraga-López P.J., Victoria-Montesinos D. (2023). Global proportion of disordered eating in children and adolescents: A systematic review and meta-analysis. JAMA Pediatr..

[10] Jahrami H., Sater M., Abdulla A., Faris M.E.A.I., AlAnsari A. (2019). Eating disorders risk among medical students: A global systematic review and meta-analysis. Eat. Weight Disord..

[11] Budhiwianto S., Bennett C.J., Bristow C.A., Dart J. (2023). Global prevalence of eating disorders in nutrition and dietetic university students: A systematic scoping review. Nutrients.

[12] Torres-Soto N.Y., Pérez-Zepeda B., Aguilar-Navarro Y.A., Corrales-Baldenebro A.L., Martínez-Ramírez B. (2026). The association between academic stress and perceived academic impairment: Eating behaviors as indirect pathways in Health Sciences students. Psychol. Int..

[13] Arcelus J., Witcomb G.L., Mitchell A. (2014). Prevalence of eating disorders amongst dancers: A systemic review and meta-analysis. Eur. Eat. Disord. Rev..

[14] Silverii G.A., Benvenuti F., Morandin G., Ricca V., Monami M., Mannucci E., Rotella F. (2022). Eating psychopathology in ballet dancers: A meta-analysis of observational studies. Eat. Weight Disord..

[15] Picard M., Gaetz M. (2025). Cyclic patterns of high-risk behaviours within ballet culture. BMC Psychol..

[16] Foscolou A., Koutoulogenis K., Amerikanou C., Chatzistamatiou C., Stergiou E.M., Katsari R., Perrea A., Panoutsopoulos G.I., Kaliora A.C., Gioxari A. (2026). Eating attitudes and anthropometric indices of adult women in performance dance: A case-control study in Greece. Eur. Eat. Disord. Rev..

[17] Berengüí R., Angosto S., Hernández-Ruiz A., Rueda-Flores M., Castejón M.A. (2024). Body image and eating disorders in aesthetic sports: A systematic review of assessment and risk. Sci. Sports..

[18] Parker E., Mitchison D., Hronis A. (2026). Intrinsic and extrinsic risk factors for mental health difficulties in dancers: A systematic review. J. Danc. Med. Sci..

[19] Ryterska K., Kulczynska P. (2025). Predisposition to eating disorders among ballet dancers: An analysis using the EAT-26 and EDE-Q questionnaires. Cent. Eur. J. Sport Sci. Med..

[20] Penniment K.J., Egan S.J. (2012). Perfectionism and learning experiences in dance class as risk factors for eating disorders in dancers. Eur. Eat. Disord. Rev..

[21] Radell S.A., Mandradjieff M.P., Adame D.D., Cole S.P. (2020). Impact of mirrors on body image of beginning modern and ballet students. J. Danc. Med. Sci..

[22] Valverde M.G., de Oliveira N.C.F., de Matos B.P., de Vasconcelos C.O., Salzer E.B., Chaves B.S., Neves C.M. (2026). The Body Between Technique and Aesthetics Eating Disorders in Dance.

[23] Gorrell S., Sanzari C.M., Ziegenhardt P.S., Spell J., Johnson M.G., Whitlow B., Saigal M. (2024). Preventing eating pathology among elite adolescent ballet dancers: A pilot trial of the Athlete Body Project. Eat. Disord..

[24] Thomas J.J., Keel P.K., Heatherton T.F. (2005). Disordered eating attitudes and behaviors in ballet students: Examination of environmental and individual risk factors. Int. J. Eat. Disord..

[25] Di Gesto C., Matera C., Policardo G.R., Nerini A. (2023). Instagram as a digital mirror: The effects of instagram likes and disclaimer labels on self-awareness, body dissatisfaction, and social physique anxiety among young Italian women. Curr. Psychol..

[26] Doyle-Lucas A.F., Davy B.M. (2011). Development and evaluation of an educational intervention program for pre-professional adolescent ballet dancers: Nutrition for optimal performance. J. Danc. Med. Sci..

[27] Blevins P., Erskine S., Hopper L., Moyle G. (2020). Finding your balance: An investigation of recovery–stress balance in vocational dance training. J. Danc. Educ..

[28] Junge A., Hauschild A., Stubbe J.H., van Rijn R.M. (2024). Health problems of professional ballet dancers: An analysis of 1627 weekly self-reports on injuries, illnesses and mental health problems during one season. Sports Med. Open.

[29] Wulff H. (2020). Ballet Across Borders: Career and Culture in the World of Dancers.

[30] Wensel S., Kerr G., Tamminen K.A. (2024). “The abuse of minors you witness or experience”: A qualitative study of former professional ballet school students’ characterizations of maltreatment. Child Abus. Negl..

[31] Russell J.A., Armour P.J., Hodge L.L., Pereira R., Petery S.M. (2026). Analysing injuries to dancers working in the commercial dance industry. Occup. Med..

[32] Leka S., Jain A. (2024). Conceptualising Work-Related Psychosocial Risks: Current State of the Art and Implications for Research, Policy and Practice.

[33] Lupien S.J., McEwen B.S., Gunnar M.R., Heim C. (2009). Effects of stress throughout the lifespan on the brain, behaviour and cognition. Nat. Rev. Neurosci..

[34] Castellini G., Castellani W., Lelli L., Sauro C.L., Dini C., Lazzeretti L., Bencini L., Mannucci E., Ricca V. (2014). Association between resting energy expenditure, psychopathology and HPA-axis in eating disorders. World J. Clin. Cases.

[35] Lo Sauro C., Ravaldi C., Cabras P.L., Faravelli C., Ricca V. (2008). Stress, hypothalamic-pituitary-adrenal axis and eating disorders. Neuropsychobiology.

[36] Simon J.J., Stopyra M.A., Friederich H.-C. (2019). Neural processing of disorder-related stimuli in patients with anorexia nervosa: A narrative review of brain imaging studies. J. Clin. Med..

[37] Kaye W.H., Wierenga C.E., Bailer U.F., Simmons A.N., Bischoff-Grethe A. (2013). Nothing tastes as good as skinny feels: The neurobiology of anorexia nervosa. Trends Neurosci..

[38] Torres S.J., Nowson C.A. (2007). Relationship between stress, eating behavior, and obesity. Nutrition.

[39] Mountjoy M., Ackerman K.E., Bailey D.M., Burke L.M., Constantini N., Hackney A.C., Heikura I.A., Melin A., Pensgaard A.M., Stellingwerff T. (2023). 2023 International Olympic Committee’s (IOC) consensus statement on Relative Energy Deficiency in Sport (REDs). Br. J. Sports Med..

[40] Sullivan K.M. Open Source Statistics for Public Health. https://www.openepi.com/SampleSize/SSPropor.htm.

[41] Garner D.M., Olmsted M.P., Bohr Y., Garfinkel P.E. (1982). The eating attitudes test: Psychometric features and clinical correlates. Psychol. Med..

[42] Wider W., Mutang J.A., Chua B.S., Lin J., Kamu A., Pang N.T.P. (2023). Assessing the factor structure of the Eating Attitude Test-26 among undergraduate students in Malaysia. Front. Nutr..

[43] Mackay C., Cousins R., Kelly P.J., Lee S., McCaig R. (2004). ‘Management Standards’ and work-related stress in the UK: Policy background and science. Work Stress.

[44] Bacevičienė M., Jankauskienė R. (2021). The associations between young women’s perfectionism, self-objectification, and the risk of eating disorders. Soc. Res..

[45] McLean C.P., Kulkarni J., Sharp G. (2023). The 26-Item Eating Attitudes Test (EAT-26): Psychometric properties and factor structure in vegetarians and vegans. Nutrients.

[46] Nohara N., Hiraide M., Horie T., Takakura S., Hata T., Sudo N., Yoshiuchi K. (2024). The optimal cut-off score of the Eating Attitude Test-26 for screening eating disorders in Japan. Eat. Weight Disord..

[47] Spivak-Lavi Z., Peleg O., Tzischinsky O., Stein D., Latzer Y. (2021). Differences in the factor Structure of the Eating Attitude Test-26 (EAT-26) in different cultures in Israel: Jews, Muslims, and Christians. Nutrients.

[48] Guidi S., Bagnara S., Fichera G.P. (2012). The HSE indicator tool, psychological distress and work ability. Occup. Med..

[49] Strukcinskiene B., Jurgaitis J., Grigoliene R., Karoblyte D., Zuperkiene E., Martisauskiene D., Gedvile Z., Virketis G., Venclauskas L., Genowska A. (2025). Patterns of self-reported occupational stress experienced by Lithuanian police officers: A cross-sectional study. Healthcare.

[50] Kuodytė V., Pajarskienė B. (2017). Psychometric properties of the Lithuanian version of the HSE Stress Indicator Tool. Public Health.

[51] Health and Safety Executive (HSE) (2019). HSE Management Standards Indicator Tool User Manual.

[52] Tang D., Chen M., Huang X., Zhang G., Zeng L., Zhang G., Wu S., Wang Y. (2023). SRplot: A free online platform for data visualization and graphing. PLoS ONE.

[53] Bahl E., Koomar T., Michaelson J.J. (2017). CerebroViz: An R package for anatomical visualization of spatiotemporal brain data. Bioinformatics.

[54] Miller J.A., Ding S.L., Sunkin S.M., Smith K.A., Ng L., Szafer A., Ebbert A., Riley Z.L., Royall J.J., Aiona K. (2014). Transcriptional landscape of the prenatal human brain. Nature.

[55] Cohen J. (1988). Statistical Power Analysis for the Behavioral Sciences.

[56] Tharwat A., Gaber T., Ibrahim A., Hassanien A.E. (2017). Linear discriminant analysis: A detailed tutorial. AI Commun..

[57] Strengthening the Reporting of Observational Studies in Epidemiology (STROBE). https://www.strobe-statement.org/.

[58] Nordin-Bates S.M., Quested E., Walker I.J., Redding E. (2012). Climate change in the dance studio: Findings from the UK centres for advanced training. Sport Exerc. Perform. Psychol..

[59] Thomas J.J., Lawson E.A., Micali N., Misra M., Deckersbach T., Eddy K.T. (2017). Avoidant/restrictive food intake disorder: A three-dimensional model of neurobiology with implications for etiology and treatment. Curr. Psychiatry Rep..

[60] Ringham R., Klump K., Kaye W., Stone D., Libman S., Stowe S., Marcus M. (2006). Eating disorder symptomatology among ballet dancers. Int. J. Eat. Disord..

[61] Nazem T.G., Ackerman K.E. (2012). The female athlete triad. Sports Health.

[62] Holihan A.J. (2022). Elevating Artists’ Voices: Examining Organizational Dynamics Between Ballet Company Dancers and Leadership. Master’s Thesis.

[63] Rigoli A., Dang E., Michael V., Gifford J., Davies A. (2024). What do we know about the energy status and diets of pre-professional and professional dancers: A scoping review. Nutrients.

[64] Kelman B.B. (2000). Occupational hazards in female ballet dancers: Advocate for a forgotten population. AAOHN J..

[65] Woropay-Hordziejewicz N.A., Buźniak A., Lawendowski R., Atroszko P.A. (2022). Compulsive study behaviors are associated with eating disorders and have independent negative effects on well-being: A structural equation model study among young musicians. Sustainability.

[66] Fostervold Mathisen T.F., Sundgot-Borgen C., Anstensrud B., Sundgot-Borgen J. (2022). Mental health, eating behaviour and injuries in professional dance students. Res. Danc. Educ..

[67] Cohen S., Wills T.A. (1985). Stress, social support, and the buffering hypothesis. Psychol. Bull..

[68] Mason T.B., Lewis R.J. (2017). Examining social support, rumination, and optimism in relation to binge eating among Caucasian and African-American College women. Eat. Weight Disord..

[69] Drury C.R., Armeli S., Loeb K.L. (2024). Body checking and avoidance among dancers. Eat. Behav..

[70] Frank G.K., Shott M.E., Hagman J.O., Mittal V.A. (2013). Taste reward circuitry related brain structures characterize ill and recovered anorexia nervosa and bulimia nervosa. Am. J. Psychiatry.

[71] Borakowska D., Podgórski R., Łuszczki E. (2026). Adaptive changes in adrenal steroid metabolism under extreme physical and psychological stress: A narrative review. Front. Endocrinol..

