# Supplementary material for: Restrictive Eating Patterns and Eating Disorder Risk in Female Ballet Dancers: Exploratory Associations with Self-Reported Workplace Bullying and Possible Mobbing
Source: Nutrients. 2026 Sep 27;18(19):3191. doi: 10.3390/nu18193191 (PMC13666931; doi:10.3390/nu18193191)
Supplement: Supplementary file 1 [file nutrients-18-03191-s001.zip › nutrients-4587629-supplementary.pdf]

Supplementary Materials

Table S1. Internal consistency and item-level reliability analysis of the Eating Attitudes Test (EAT-26) scale and its subscales (Dieting, Bulimia and Food Preoccupation, and Oral Control) within the study sample (n = 377).

Item No.	The three subscales of the Eating Attitudes Test (EAT-26)	Cronbach's alfa (α)
Dieting subscale (Factor D)		0.881
Q1:	Am terrified about being overweight.	0.870
Q6:	Aware of the calorie content of foods that I eat.	0.875
Q7:	Particularly avoid food with a high carbohydrates content (i.e. bread, rice, potatoes, etc.).	0.876
Q10:	Feel extremely guilty after eating.	0.863
Q11:	Am preoccupied with a desire to be thinner.	0.866
Q12:	Think about burning up calories when I exercise.	0.869
Q14:	Am preoccupied with the thought of having fat on my body.	0.870
Q17:	Eat diet foods.	0.874
Q22:	Feel uncomfortable eating sweets.	0.863
Q23:	Engage in dieting behavior.	0.872
Q24:	Like my stomach to be empty.	0.869
Q26:	Enjoy trying new rich foods.	0.895
Bulimia and Food Preoccupation subscale (Factor B)		0.810
Q3:	Find myself preoccupied with food.	0.865
Q4:	Have gone on eating binges where I felt that I may not be able to stop.	0.854
Q9:	Vomit after I have eaten.	0.819
Q18:	Feel that food controls my life.	0.872
Q21:	Give too much time and thought to food.	0.857
Q25:	Have the impulse to vomit after meals.	0.897
Oral Control (Factor O)		0.859
Q2:	Avoid eating when I am hungry.	0.841
Q5:	Cut my food into small pieces.	0.843
Q8:	Feel that others would prefer if I ate more.	0.841
Q13:	Other people think that I am too thin.	0.854
Q15:	Take longer than others to eat my meals.	0.842
Q16:	Avoid foods with sugar in them.	0.818
Q19:	Display self-control around food.	0.811
Q20:	Feel that others pressure me to eat.	0.819
	Total	0.918

Data represent Cronbach's alpha (α) coefficients computed for the total scale, individual subscales, and each item if deleted. The analysis is based on the pooled sample comprising both professional ballet dancers and the comparison higher education student cohorts. EAT-26—Eating Attitudes Test-26.

Table S2. Internal consistency and item-level reliability analysis of the 35-item Health and Safety Executive (HSE) Indicator Tool and its seven core subscales within the professional ballet dancer cohort (*n* = 117).

Item No.	The seven subscales of the Health Service Executive (HSE) Indicator Tool	Abbrevation	Cronbach's alfa (α)
Demands subscale			0.869
Q3:	Different groups at work demand things from me that are hard to combine.	'Competing priorities	0.855
Q6:	I have unachievable deadlines.	Unrealistic deadlines	0.851
Q9:	I have to work very intensively.	Demanding work	0.855
Q12:	I have to neglect some tasks because I have too much to do.	Work in progress limit	0.837
Q16:	I am unable to take sufficient breaks.	Break deprivation	0.821
Q18:	I am pressured to work long hours.	Mandatory overtime	0.826
Q20:	I have to work very fast.	Working rapidly	0.831
Q22:	I have unrealistic time pressures.	Extreme time pressure	0.810
Control subscale			0.886
Q2:	I can decide when to take a break.	On-demand breaks	0.878
Q10:	I have a say in my own work speed.	Schedule autonomy	0.809
Q15:	I have a choice in deciding how I do my work.	Self-directed work	0.805
Q19:	I have a choice deciding what I do at work.	Decision-making power at work	0.835
Q25:	I have some say over the way I work.	High job control	0.842
Q30:	My work time can be flexible.	Flexible schedule	0.834
Managerial support subscale			0.908
Q8:	I am given supportive feedback on the work I do.	Supportive feedback on the work	0.807
Q23:	I can rely on my line manager to help me out with a work problem.	Management support	0.871
Q29:	I can talk to my line manager about something that has upset or annoyed me about work.	Possibility of complaining	0.852
Q33:	I am supported through emotionally demanding work.	Supporting emotional buffering	0.861
Q35:	Line manager encourages me at work.	Manager's encouragement	0.860
Peer support subscale			0.882
Q7:	If work gets difficult, my colleagues will help me.	Assistance from coworkers	0.886
Q24:	I get help and support I need from colleagues.	Positive peer support	0.893
Q27:	I receive the respect at work I deserve from my colleagues.	Occupational respect	0.811
Q31:	My colleagues are willing to listen to my work-related problems.	Sounding board	0.805
Relationship subscale			0.877
Q5:	I am subject to personal harassment in the form of unkind words or behavior.	Possible mobbing	0.864
Q14:	There are friction or anger between colleagues.	Strained workplace relationship	0.848
Q21:	I am subject to bullying at work.	Bullying	0.812
Q34:	Relationship at work are strained.	Difficult working relationship	0.886
Role subscale			0.882
Q1:	I am clear what is expected of me at work.	Role clarity	0.884

Q4:	I know how to go about getting my job done.	Competent	0.832
Q11:	I am clear what my duties and responsibilities are.	Clear scope of work	0.807
Q13:	I am clear about the goals and objectives for my department.	Agreeing on work goals	0.803
Q17:	I understand how my work fits into the overall aim of the organization.	Organizational line of sight	0.869
Change subscale			0.882
Q26:	I have sufficient opportunities to question managers about change at work.	Active stakeholder engagement	0.866
Q28:	Staff are always consulted about change at work.	Collaborative Change	0.833
Q32:	When changes are made at work, I am clear how they will work out in practice.	Practical understanding	0.880
	Total		0.896

Data represent Cronbach's alpha (α) coefficients computed for the total scale (Total Cronbach's α = 0.926), the seven individual subscales (bolded values), and for each specific item if deleted. The abbreviated item markers indicate the core psychosocial working condition dimensions assessed. HSE—Health and Safety Executive.
